# Variations in autologous neutralization and CD4 dependence of b12 resistant HIV-1 clade C *env *clones obtained at different time points from antiretroviral naïve Indian patients with recent infection

**DOI:** 10.1186/1742-4690-7-76

**Published:** 2010-09-22

**Authors:** Rajesh Ringe, Madhuri Thakar, Jayanta Bhattacharya

**Affiliations:** 1 Department of Molecular Virology, National AIDS Research Institute, Indian Council of Medical Research, G-73 MIDC, Bhosari, Pune-411026, India

## Abstract

**Background:**

Limited information is available on HIV-1 Indian clade C sensitivities to autologous antibodies during the course of natural infection. In the present study, a total of 37 complete envelope clones (Env) were amplified at different time points predominantly from the plasma of five Indian patients with recent HIV-1 infection and envelope-pseudotyped viruses were examined for their magnitude of sensitivity to autologous plasma antibodies during natural course of infection.

**Results:**

Variable low levels of neutralization were consistently detected with contemporaneous autologous plasma. In contrast to clade B and African clade C HIV-1 envelopes, Env clones obtained from four patients were found to be resistant to IgG1b12. The majority of the Env clones were resistant to 2G12 and 2F5 due to the absence of the minimal motifs required for antibody recognition, but were sensitive to 4E10. Nonetheless, Env clones from one patient were found to be sensitive to 2G12, atypical for clade C, and one Env clone exhibited unusual sensitivity to 17b, suggesting spontaneous exposure of CD4i epitopes. Phylogenetic analysis revealed that Env clones were closely clustered within patients. Variation in the potential N-linked glycosylation pattern also appeared to be different in patients over the course of infection. Interestingly, we found that the sensitivity of Envs to contemporaneous autologous NAbs correlated positively with increased sensitivity to soluble CD4 and inversely with anti-CD4 antibody and Envs with increased NAb sensitivity were able to efficiently infect HeLa cells expressing low CD4.

**Conclusion:**

Our data showed considerable variations in autologous neutralization of these early HIV-1 clade C Envs in each of these patients and indicate greater exposure to CD4 of Envs that showed increased autologous neutralization. Interestingly, Env clones obtained from a single patient at different time points were found to retain sensitivity to b12 antibody that binds to CD4 binding site in Env in contrast to Envs obtained from other patients. However, we did not find any association between increased b12 sensitivity of Envs obtained from this particular patient with their degree of exposure to CD4.

## Background

Induction of broadly neutralizing antibodies (NAbs) against diverse strains of Human Immunodeficiency Virus Type 1 (HIV-1) remains an important goal for vaccine development [1-3]. Major obstacles are the remarkable sequence variability of the envelope glycoproteins (Env) and the masking of critical neutralizing epitopes by N-linked glycans and other structural and steric constraints [4-6]. Most HIV-1-infected individuals mount a strong autologous NAb response within the first 6 to 12 months of infection that is highly specific for the subject's transmitted/founder virus. The response generally broadens after several years of infection, where in approximately 10-20 percent of cases the antibodies exhibit considerable breadth of neutralization against diverse strains [7-15].

HIV-1 entry is mediated by binding of trimeric gp120 spikes to CD4 receptor that in turn exposes coreceptor binding sites and facilitates fusion of viral and cell membrane [16]. NAbs bind to exposed epitopes on Env trimers and thereby compromise HIV-1 entry [17,6,19]. The discovery of broadly neutralizing monoclonal antibodies (MAbs) from HIV-1-infected patients with the ability to neutralize diverse primary HIV-1 isolates [20-23], suggested that there are indeed vulnerable epitopes on the functional Env trimer [24]. Thus, MAb IgG1b12 binds the CD4-binding site (CD4bs) of gp120 [25] and neutralizes more than 50% of HIV-1 clade B and approximately 30% of non-clade B viruses [26,27]. Although many neutralization epitopes can be masked by N-linked glycans, one MAb, 2G12 [28,29], binds to specific glycan residue and neutralizes many clade B isolates but has limited breadth against non-clade B isolates [26,30,31]. In addition, highly conserved sequences [32] in the coreceptor binding site (also known as CD4-induced or CD4i region) are potential targets for virus neutralization [33-36]. Thus, antibodies mimicking prototype MAb 17b show significant virus neutralization after triggering gp120 with soluble CD4 (sCD4) [24]. Apart from epitopes in gp120 recognized by broadly neutralizing MAbs, the membrane proximal external region (MPER) in gp41 is vulnerable to NAbs and found to be a target of three well characterized MAbs 2F5, 4E10, and Z13 [37-39]. Antibodies targeting the MPER of gp41 neutralize HIV-1 by blocking viral fusion with the cell membrane and thereby preventing viral entry [40]. 59). Interestingly, these types of antibodies are rarely detected during natural infection [22,41,42].

Being highly variable, Env remains a major target of the NAb response in HIV-1-infected individuals; thus, Env-driven antibodies have been shown to neutralize autologous virus variants moderately over time [12,13,43,44], followed by rapid escape from neutralization. Autologous NAbs appear to be directed to variable regions of gp120 and are influenced by the pattern of surface Env glycosylation that varies widely among HIV-1 strains [9,10,44-52]. These data indicate that despite a limited role for autologous NAbs in the control of viremia, the antibodies exert selection pressure on Env early in infection. In the case of HIV-1 clade B, the V1, V2 and V3 domains have also been shown to mediate CD4 independence, cellular tropism and receptor utilization in addition to neutralization sensitivity [49,53-65].

HIV-1 clade C is the dominant genetic subtype circulating in India, Sub-Saharan Africa and China [66-70]. Though much information on autologous NAbs against HIV-1 African clade C is available [9,10,42,49,50,52,71,72], very limited information is available on the neutralization properties of subtype C HIV-1 in India. Current evidence suggests that sequences for the Indian HIV-1 clade C Env and other genes such as *gag *and *nef *form a monophyletic lineage and segregate separately as a sub clade within the more diverse subtype C strains from Africa [69,73-77]. Recently, Kulkarni *et al *[27] demonstrated that newly transmitted Indian Envs are antigenically complex despite close genetic similarity. In this paper, we examined the NAb response in subtype C HIV-1-infected individuals in India by using Env clones amplified from uncultured peripheral blood mononuclear cells (PBMC) at the baseline, and plasma at the follow up visits of five recently infected subjects and assessed autologous NAbs at different time points for one year. We found that patient Envs varied considerably in their sensitivities to their autologous plasma antibodies and differed in their susceptibilities to MAbs, indicating distinct mechanisms of autologous neutralization and antibody specificities in these patients.

## Results

### Genetic properties of clade C *env *clones

**Figure 1:**
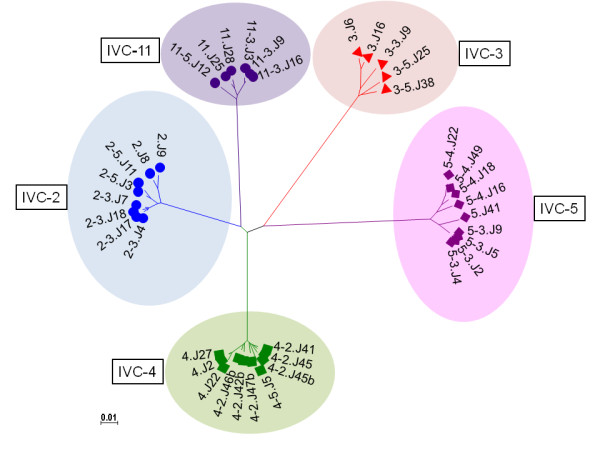
**Phylogenetic relationships between inter and intra-patient Env gp160 amino acid sequences used to study virus neutralization as determined by Neighbor-Joining maximum likelihood tree using Mega 4.1**. Bootstrapped values indicated that Env sequences were patient specific and indicated monophyletic clustering of intra-patient Env.

**Figure 2:**
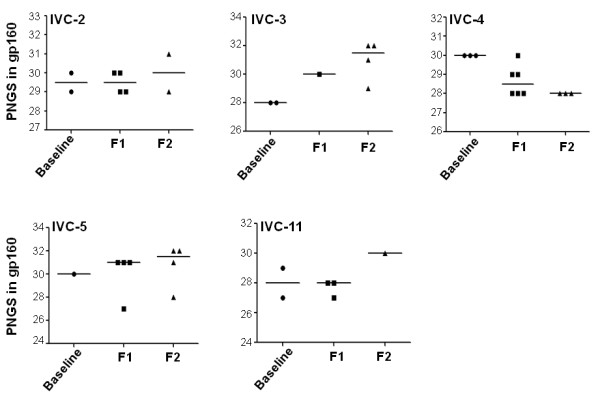
**Variations of PNLGs in patient Envs at different time points during the course of infection**. The bar represents median values.

**Figure 3:**
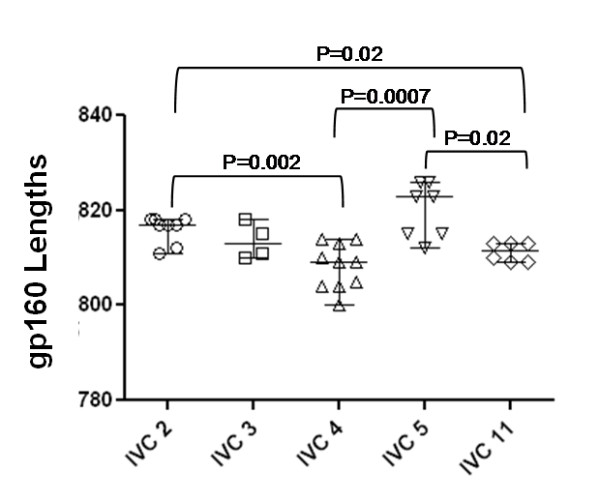
**Variations in total gp160 lengths of Env clones obtained at different times in each patient during the course of infection**. Each bar represents median gp160 residues. Note that significant differences in median gp160 lengths of Envs between IVC 2 and 4, IVC 2 and 11, IVC 4 and 5 and IVC 5 and 11.

Study subjects are described in Table 1. More than one *env *clones was obtained from each of five recently infected HIV-1 positive individuals from India at a baseline visit and 6 and 12 months later except for subject IVC5, for whom the last visit was at 24 months (Table 2). Env clones from the baseline visit were obtained from infected PBMC DNA whereas for follow up visits, env was amplified from plasma viral RNA. Phylogenetic analyses of the complete gp160 amino acid sequences revealed that the Env clones were indeed subject specific (Figure 1), with intra-clonal genetic divergences between Env clones obtained from the same subject but at different time points indicated ongoing viral evolution. All Envs possessed low net V3 loop charge, a conserved GPGQ motif (Additional file 1: Figure S1) and were found to be CCR5 tropic (Table 2). Except for patients IVC 3 and IVC 4, no significant variation in total N-linked glycosylation sites (PNLG) was found at the three time points sampled (Figure 2); the number of PNLG varied between 25-31 (Table 2). Median gp160 lengths varied between patients; however they did not differ significantly between clones obtained from the same patient at different times (Figure 3). Although there were no major differences between the variable loops of the patient-specific envelope clones obtained at different time points, Env clones 3-3.J9, 3-5.J25 and 5-4.J49, 5-4.J16 amplified from patients IVC 3 and IVC 5 were found to have shorter V1 and V2 loops compared to the contemporaneous Env clones (Additional file 1: Figure S1).

**Table 1:** Patient details

			Plasma HIV-1 RNA (copies/ml)			CD4 count (cells/mm3)		
			
Patient ID	Mode of Transmission	Year of Infection	Baseline	F1 (moths)	F2 (months)	Baseline	F1 (months)	F2 (months)
NARI-IVC-2	Heterosexual	2008	8400	3070 (6)	17700 (12)	479	503 (6)	135 (12)

NARI-IVC-3	Heterosexual	2006	5380	29700 (6)	15700 (12)	592	499 (6)	477 (12)

NARI-IVC-4	Heterosexual	2006	37800	UD (6)	UD (12)	328	374 (6)	402 (12)

NARI-IVC-5	Heterosexual	2006	1410	9040 (6)	48600 (24)	606	619 (6)	427 (24)

NARI-IVC-11	Heterosexual	2007	33400	11900 (6)	17300 (12)	552	693 (6)	590 (12)

UD: undetermined

**Table 2:** Genetic properties of patient Env clones

Patient ID	Clone ID/Follow up Schedule†	Source	gp120 length	gp41 length	PNLG sites	Net V3 loop charge	CoR usage
	2.J8/B	PBMC	466	352	25	3	CCR5
	
	2.J9/B	PBMC	466	352	26	3	CCR5
	
	2-3.J4/F1	PLASMA	465	352	30	3	CCR5
	
NARI-IVC2	2-3.J7/F1	PLASMA	466	352	29	3	CCR5
	
	2-3.J17/F1	PLASMA	460	352	28	3	CCR5
	
	2-3.J18/F1	PLASMA	465	352	30	3	CCR5
	
	2-5.J3/F2	PLASMA	466	345	31	3	CCR5
	
	2-5.J11/F2	PLASMA	465	352	29	2	CCR5

	3.J16/B	PBMC	466	352	27	5	CCR5
	
NARI-IVC3	3-3.J9/F1	PLASMA	459	352	28	5	CCR5
	
	3-5.J25/F2	PLASMA	458	352	29	4	CCR5
	
	3-5.J38/F2	PLASMA	463	352	31	3	CCR5

	4.J2/B	PBMC	462	352	30	3	CCR5
	
	4.J22/B	PBMC	462	352	30	3	CCR5
	
	4.J27/B	PLASMA	461	352	29	3	CCR5
	
	4-2.J41/F1	PLASMA	458	352	27	2	CCR5
	
	4-2.J45/F1	PLASMA	460	345	27	2	CCR5
	
NARI-IVC4	4-2.J42b/F1	PLASMA	464	345	27	2	CCR5
	
	4-2.J45b/F1	PLASMA	459	345	26	2	CCR5
	
	4-2.J46b/F1	PLASMA	464	345	28	2	CCR5
	
	4-2.J47b/F1	PLASMA	459	345	27	2	CCR5
	
	4-5.J5/F2	PLASMA	455	345	28	2	CCR5

	5.J41/B	PBMC	472	351	29	2	CCR5
	
	5-3.J2/F1	PLASMA	461	351	26	3	CCR5
	
	5-3.J4/F1	PLASMA	472	351	29	3	CCR5
	
	5-3.J5/F1	PLASMA	461	362	30	3	CCR5
	
	5-3.J9/F1	PLASMA	472	351	29	3	CCR5
	
NARI-IVC5	5-4.J16/F2	PLASMA	464	351	31	3	CCR5
	
	5-4.J18/F2	PLASMA	475	351	30	4	CCR5
	
	5-4.J22/F2	PLASMA	464	351	28	3	CCR5
	
	5-4.J49/F2	PLASMA	475	351	30	3	CCR5

	11.J25/B	PBMC	461	352	27	4	CCR5
	
	11.J28/B	PBMC	461	352	27	4	CCR5
	
	11-3.J3/F1	PLASMA	458	352	28	4	CCR5
	
NARI-IVC11	11-3.J9/F1	PLASMA	457	352	27	4	CCR5
	
	11-3.J16/F1	PLASMA	457	352	26	4	CCR5
	
	11-5.J12/F2	PLASMA	461	352	28	3	CCR5

† B = Baseline sample; F1 = First Follow up and F2 = Second follow up.

### Neutralization sensitivity of clonal Envs to autologous plasma varied between study subjects

We next assessed the autologous neutralization of Env clones amplified at three different time points from each of five subjects. All five subjects mounted a moderate NAb response against their early viruses obtained at the baseline except patient IVC2; however this phenotype varied with respect to contemporaneous plasma antibodies (Table 3). Surprisingly, only 1/8 clones from subject IVC-2 was neutralized by the plasma samples obtained at later time points, whereas a few (3/8) Env clones were neutralized by the contemporaneous plasma. Thus, while the autologous NAb response to early Env clones improved over time in four subjects, it diminished over time in one subject. This observation was correlated with a gradual decline in CD4, indicating that autologous NAb possibly has selected the fittest Env variants capable of faster disease progression in this particular patient. The majority of the Envs obtained from follow up visits were resistant to contemporaneous autologous plasma antibodies indicating rapid escape of viral variants. The persistence of a few sensitive Envs such as 3-3.J9/F1, and 4-2.J45 during this period of infection despite mounting humoral immune pressure may indicate that these Env variants had adapted to sustain such immune pressure possibly through certain compensatory changes in Env sequence and retained their sensitivities to autologous neutralizing antibodies.

**Table 3:** Neutralization sensitivity of patient envelopes to autologous plasma antibodies

*Env *clones	Baseline plasma	Plasma First visit (F1)	Plasma Second visit (F2)
2.J8	601	228	<20

2.J9	522	240	<20

2-3.J4	350	<20	<20

2-3.J7	374	<50	<20

2-3.J17	300	<20	<20

2-3.J18	<20	540	652

2-5.J3	<50	<20	<20

2-5.J11	50	<20	<20

3.J16	195	696	2389

3-3.J9	349	554	1053

3-5.J25	<20	<20	184

3-5.J38	<20	<20	72

4.J2	421	2671	3848

4.J22	87	811	1172

4.J27	74	773	871

4-2.J41	103	98	406

4-2.J45	3375	6287	8307

4-2.J42b	60	<20	115

4-2.J45b	70	<50	500

4-2.J46b	<50	<50	160

4-2.J47b	72	<50	340

4-5.J5	64	<20	244

5.J41	<20	110	1934

5-3.J2	<20	<20	1845

5-3.J4	<20	<20	1067

5-3.J5	<20	<20	1161

5-3.J9	<20	<20	1104

5-4.J16	<20	<20	<50

5-4.J18	<20	<20	<50

5-4.J22	<20	<50	223

5-4.J49	<20	<50	180

11.J25	66	2158	2830

11.J28	76	2008	2310

11-3.J3	<20	<50	1193

11-3.J9	<20	<20	148

11-3.J16	<20	<20	201

11-5.J12	<20	<20	<50

Values are reciprocal titer of patient plasma resulting 50% reduction in relative luminescence unit (RLU) as an indicator of neutralization sensitivity in TZM-bl cells following infection with pseudoviruses with 200TCID_50_. The ID_50 _values are average of two independent assays wherein each assay was done in duplicates.

### Neutralization phenotype of the Envs as assessed with common neutralizers

To test if the Envs obtained from patients at different time points varied in their sensitivities to common broadly neutralizing MAbs, pseudotyped viruses carrying patient Envs were tested in neutralization assays with sCD4 and five MAbs (b6, IgG1b12, 2G12 targeting gp120 and 2F5, and 4E10 targeting gp41). As shown in Table 4 the majority of Env clones were sensitive to sCD4 at concentrations ranging from 0.1 to 6.66 μg/ml. The pseudoviruses that required excess (>6.66 μg/ml) sCD4 for 50% neutralization were considered as resistant in our study. Consistent with the earlier report [27] all Env variants were resistant to 2G12 except those obtained from IVC-3 patient and this resistance was associated with the absence of PNLG at position 295 (HXB2 numbering) at the N-terminal base of V3 loop. The sensitivity of IVC-3 env clones was due to the presence of N295, atypical of clade C. In contrast to clade B and African clade C viruses [10,26], envelopes from patient IVC 3, 4, 5, 11 were found resistant to IgG1b12. This observation of b12 resistance of the India clade Envs is in line with that reported by Kulkarni *et al *[27]. As with the MPER-specific MAbs, all the Envs were resistant to 2F5 at the highest concentration tested (Table 4). Interestingly, while 2F5 resistance was found to be associated with the absence of DKW motif in gp41 in most of the Envs, this motif was found to be present in IVC3-3-9F1, IVC3-5-25F2, and all the Envs obtained from IVC-11 and conferred resistance as shown in Additional file 2: Table S1. Our data indicate that residues outside MPER domain possibly modulated 2F5 sensitivity despite the presence of a minimum DKW motif in MPER for 2F5 sensitivity. The ability of 4E10 to neutralize all the env clones was in agreement with the presence of WFXI motif in gp41; however 4 Envs (4-2_NEM.J46b, 4-5_NEM.J5, 5-3_NEM.J4 and 5-3_NEM.J9) despite having WFXI motif (a minimum 4E10 recognition motif), they were found to be moderately resistant to 4E10 up to a concentration of 6.66 μg/ml (Additional file 1: Figure S1 and Additional file 2: Table S1).

**Table 4:** Neutralization sensitivity to monoclonal antibodies, sCD4 and anti-CD4

*Env *clones	b6	b12	2G12	17b	2F5	4E10	sCD4	SIM.2*
2.J8	>6.66	2.23	>6.66	>6.66	>6.66	0.34	3.66	120

2.J9	>6.66	2.16	>6.66	>6.66	>6.66	0.38	3.27	104

2-3.J4	>6.66	1.97	>6.66	>6.66	>6.66	3.36	>6.66	260

2-3.J7	>6.66	2.19	>6.66	>6.66	>6.66	5.85	>6.66	260

2-3.J17	>6.66	2.04	>6.66	>6.66	>6.66	4.85	>6.66	106

2-3.J18	>6.66	0.5	>6.66	5.1	>6.66	4.5	>6.66	37

2-5.J3	>6.66	0.29	>6.66	>6.66	>6.66	2.69	>6.66	201

2-5.J11	>6.66	0.21	>6.66	>6.66	>6.66	0.32	6.05	152

3.J16	>6.66	>6.66	4.20	>6.66	>6.66	0.23	0.54	103

3-3.J9	>6.66	>6.66	0.18	2.9	>6.66	0.3	0.1	76

3-5.J25	>6.66	>6.66	4.85	>6.66	>6.66	2.6	3.3	106

3-5.J38	>6.66	>6.66	4.30	>6.66	>6.66	2.22	>6.66	79

4.J2	>6.66	>6.66	>6.66	>6.66	>6.66	0.28	0.5	10

4.J22	>6.66	>6.66	>6.66	>6.66	>6.66	4	>6.66	138

4.J27	>6.66	>6.66	>6.66	>6.66	>6.66	5.28	>6.66	142

4-2.J41	>6.66	>6.66	>6.66	>6.66	>6.66	2.64	2.28	164

4-2.J45	>6.66	>6.66	>6.66	>6.66	>6.66	3.94	2.53	50

4-2.J42b	>6.66	>6.66	>6.66	>6.66	>6.66	5	>6.66	224

4-2.J45b	>6.66	>6.66	>6.66	>6.66	>6.66	6.2	>6.66	265

4-2.J46b	>6.66	>6.66	>6.66	>6.66	>6.66	>6.66	>6.66	240

4-2.J47b	>6.66	>6.66	>6.66	>6.66	>6.66	6.5	>6.66	212

4-5.J5	>6.66	>6.66	>6.66	>6.66	>6.66	>6.66	>6.66	334

5.J41	>6.66	>6.66	>6.66	>6.66	>6.66	0.29	0.5	114

5-3.J2	>6.66	>6.66	>6.66	>6.66	>6.66	5.6	>6.66	119

5-3.J4	>6.66	>6.66	>6.66	>6.66	>6.66	>6.66	>6.66	119

5-3.J5	5.9	>6.66	>6.66	>6.66	>6.66	5.66	>6.66	210

5-3.J9	>6.66	>6.66	>6.66	>6.66	>6.66	>6.66	>6.66	222

5-4.J16	>6.66	>6.66	>6.66	>6.66	>6.66	2.32	2.94	320

5-4.J18	>6.66	>6.66	>6.66	>6.66	>6.66	2.52	>6.66	157

5-4.J22	2.5	>6.66	>6.66	>6.66	>6.66	0.24	0.23	44

5-4.J49	5.9	>6.66	>6.66	>6.66	>6.66	0.52	0.53	121

11.J25	>6.66	>6.66	>6.66	>6.66	>6.66	0.34	3	99

11.J28	>6.66	>6.66	>6.66	>6.66	>6.66	0.32	2.4	88

11-3.J3	>6.66	>6.66	>6.66	>6.66	>6.66	5.64	>6.66	548

11-3.J9	>6.66	>6.66	>6.66	>6.66	>6.66	3.35	>6.66	555

11-3.J16	>6.66	6.05	>6.66	>6.66	>6.66	3.25	>6.66	585

11-5.J12	>6.66	>6.66	>6.66	>6.66	>6.66	2.67	>6.66	571

Values are concentrations resulting 50% reduction in relative luminescence unit (RLU) as an indicator of neutralization sensitivity in TZM-bl cells following infection with pseudoviruses with 200TCID_50_. The IC_50 _values are average of two independent assays wherein each assay was done in duplicates. * The values corresponding to anti-CD4 SIM.2 is hybridoma fluid are reciprocal dilutions giving 50% reduction in relative luminescence unit (RLU).

### Envs from one patient (NARI-IVC2) were moderately sensitive to IgG1b12 but were resistant to contemporaneous plasma antibodies

**Figure 4:**
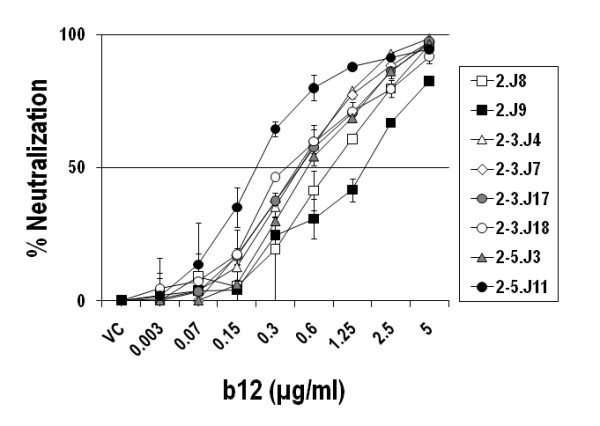
**Sensitivity of Env clones amplified from IVC2 patients to IgG1b12 antibody**. Env-pseudotyped viruses were incubated with IgG1b12 at indicated concentrations for 1 hour before TZM-bl cells were added as described in the Methods. The reduction of infectivity of TZM-bl cells was measured as a function of the degree of IgG1b12 mediated neutralization of these Envs.

In contrast all others, Envs amplified from a patient (NARI-IVC2) showed reasonable sensitivity to b12 MAb that targets CD4bs in Env. As shown in Figure 4, these Envs were found to provide a 50% reduction in infection in TZM-bl cells at concentrations ranging from 0.2 to 2.23 μg/ml. The extent of b12 sensitivities of Envs obtained from this particular patient were found to be much higher than the two b12-sensitive Indian clade C Envs reported by Kulkarni *et al *[27]. The degree of b12 sensitivity of IVC Envs, however, did not correlate with their sensitivity to sCD4 and contemporaneous plasma antibodies. Thus, Envs 2-3.J18, 2-5.J3 and 2-5.J11 which showed the highest neutralization sensitivity (IC_50 _of 0.5, 0.29 and 0.21 μg/ml respectively) to b12 required more sCD4 for 50% neutralization and except for 2-3.J18 showed neutralization resistance to contemporaneous plasma antibodies (Tables 3 and 4). Our data indicated that escape from contemporaneous NAbs in turn mounted structural constraints in Env specifically on CD4 binding site. This feature therefore possibly contributed in reduced sensitivity of NAb resistant IVC2 envelopes to sCD4, although all envelopes in this patient surprisingly retained b12 sensitivity.

### Sensitivity of Envs to contemporaneous autologous NAbs correlated positively with increased sensitivity to sCD4 and inversely with anti-CD4 antibody

**Figure 5:**
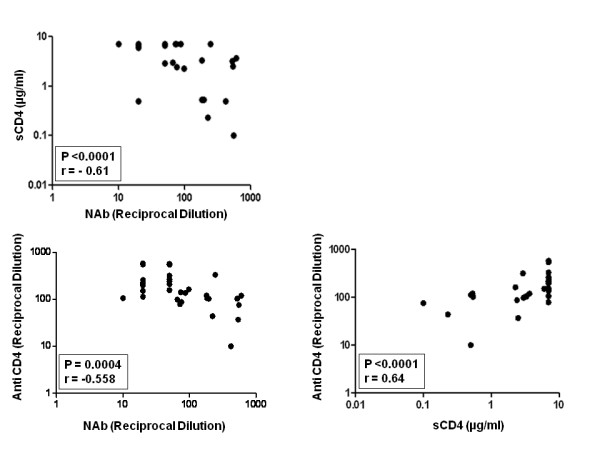
**Correlations between autologous neutralization sensitivities of patient Envs with their relative susceptibilities to sCD4 and anti-CD4 antibody (SIM.2)**. Note that Envs that required sCD4 more than 6.66 μg/ml were given a value of 7 μg/ml for the benefit of calculation. A strong correlation was observed between autologous neutralization and Env sensitivity to sCD4 (P < 0.0001) and SIM.2 (P = 0.0004) and between Env susceptibilities to sCD4 and anti-CD4 (P =< 0.0001).

To assess whether the increased sensitivity of patient envelopes to autologous NAbs could be due to greater flexibilities of gp120 interactions with CD4, we next compared the sensitivities of patient Envs to autologous plasmas, sCD4 and an anti-CD4 monoclonal antibody (SIM.2) (hybridoma supernatant) that blocks gp120-CD4 binding. Interestingly, Envs that were resistant to contemporaneous plasmas were less sensitive to sCD4 and required less anti-CD4 antibody (SIM.2) for 50% inhibition. Thus, as shown in Figure 5, a positive association was seen between Env sensitivity to contemporaneous autologous plasma and an increased sensitivity to sCD4 and inverse correlation between Env sensitivity to autologous NAb anti-CD4 antibody, suggesting that Envs with increased sensitivities to sCD4 exhibited greater exposure of epitopes than are targeted by autologous antibodies. The reduced sensitivity of Envs to SIM.2 suggests that Envs with more exposed epitopes for sCD4 require more anti-CD4 antibody for optimum inhibition to entry. Overall, the sensitivities of Envs to sCD4 varied and inversely correlated with their inhibition by SIM.2.

### Increased sensitivity of patient Envs to contemporaneous NAb and sCD4 correlated with reduced CD4 dependence

**Figure 6:**
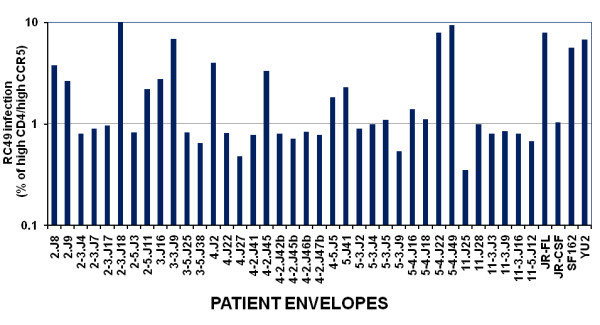
**Variation in CD4-dependence of pseudoviruses carrying patient Envs**. Pseudoviruses carrying distinct patient Envs were used to infect HeLa cells (RC49 cell line) and the infectivity expressed as percentage infection of these pseudoviruses that infected HeLa cells expressing high CD4 and high CCR5 (JC53 cell line).

**Figure 7:**
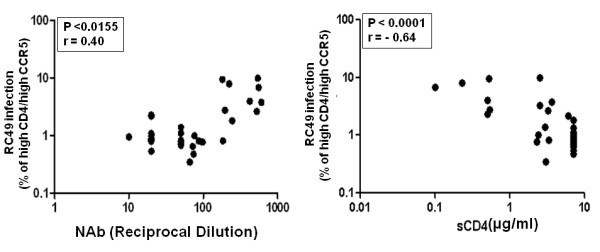
**Correlation between CD4 dependence of patient Envs with their sensitivities to autologous plasma antibodies and sCD4**. Association of CD4 usage of Env-pseudotyped viruses with autologous plasma antibodies and (P < 0.0155) and sCD4 (P < 0.0001) indicated that Env-pseudotyped viruses with low CD4 dependence tend to be more susceptible to autologous NAb in the patients tested here.

We next investigated if Envs with increased sensitivity to autologous antibodies and sCD4 exhibited greater binding to cell surface CD4. Thus, HeLa cells expressing low CD4 but high CCR5 (RC49 cell line) were infected with Env-pseudotyped viruses and the degree of infection was obtained by measuring the intracellular p24. As shown in Figure 6, Envs with increased sensitivity to autologous NAbs (such as 2-3.J18, 3-3.J9, 4.J2, 4-2.J45, 5-4.J22 and 5-4.J49) showed reduced CD4 dependence. However, this phenomenon was found to be independent of the patients and the follow up times examined here (Additional file 3: Figure S2). As expected, we found that increased sensitivity of Envs to autologous NAbs was correlated with reduced CD4 dependence (P < 0.0155) and increased susceptibility to sCD4 (P < 0.0001) (Figure 7). Collectively, our data showed an inverse association of autologous neutralization sensitivity of patient Envs with CD4 dependence.

## Discussion

In contrast to the HIV-1 neutralization properties of African clade C, there is only one report on the neutralization properties of HIV-1 clade C Env clones amplified from co-cultured PBMCs of acutely infected Indian patients [27]. One of the disadvantages in obtaining Env clones from co-culture is that it would potentially select for virus variants that become adapted for favorable replication in the absence of any immune pressure *in vitro*. This process would therefore fail to select viruses growing *in vivo *which are responsible for the pathogenesis in the natural course of infection. In the present study, we characterized for the first time the autologous NAb response in subtype C HIV-1 infected Indian patients using multiple molecular Env clones amplified without culture from each study subject. We found that while moderate NAb responses developed in three subjects (IVC 3, 4 and 11), no significant NAb response was detected at all three time points against contemporaneous autologous virus in the remaining two subjects (IVC 5 and IVC 11). In agreement with previous reports, as with both subtype B and African subtype C Envs, we found that in four patients (IVC3, 4, 5 and 11), Envs obtained at baseline and earlier time points were neutralized by plasma antibodies obtained at later time points, indicating repeated cycles of escape [45,52]. Of potential interest, Env clones obtained at all time points from IVC2 patient were moderately sensitive to IgG1b12, whereas Env clones from the remaining subjects were resistant to this MAb. Surprisingly NAb response in this patient waned over the period of time as plasma from later time points failed to neutralize many contemporaneous as well as earlier envelopes. Intriguingly, no correlation was observed between b12 sensitivity and sCD4 sensitivity as the b12 epitope overlaps CD4 binding site. One plausible explanation for this observation could be that this patient did not develop b12 like antibodies and possibly the absence of selective pressure on the b12 binding site caused the high sensitivity of these envelopes from IVC-2 towards b12. It was also possible that due to lack of co- evolution of b12 and other CD4 binding sites in Env, we did not find any association between b12 and sCD4 sensitivities to Env clones obtained from this particular patient. These observations indicate the presence of compensatory amino acid residues in the IVC-2 Env clones positioned either in the CD4bs or in the proximity that favored enhanced neutralization by b12 MAb. It would be important to further investigate the Env sequence that modulated b12 sensitivity in this patient.

Although we found repeated cycles of escape from autologous NAbs in all the patients, one Env variant (4-2_NEM.J45) obtained from patient NARI-IVC4 at the first follow up retained unusually high sensitivity to contemporaneous and earlier and follow-up plasmas with a mean ID_50 _of greater than 1: 3000. The persistence of this sensitive Env against which high titer of NAb was developed for at least 6 months makes this envelope interesting; in particular retention of neutralizing epitopes under immense humoral immune pressure probably indicates that this envelope might be more fit in terms of CTL pressure or increased infectivity to compensate for increased sensitivity to NAbs as previously described by Moore *et al *[45,52]. When tested against common HIV-1 neutralizing MAbs, most Envs obtained at different time points from all the five participants were resistant to IgGb6, IgG1b12, 2G12 and 2F5 and sensitive to 4E10 only. Intriguingly, two Env variants each from subjects IVC4 and IVC5 despite containing the minimum WFXI motif in gp41 MPER domain for 4E10 recognition, were found to require 4E10 antibody in excess (>6.66 μg/ml) of that required to provide 50% neutralization compared to all other Envs. Nakamura *et al *[78] recently showed that while F673N and W680G confers 4E10 resistance of HIV-1 envelopes, W680R showed variable 4E10 resistance. In all cases, IC_50 _values were reported to be in the range of greater than 50-100 μg/ml. In our study, we did not find any of these substitutions in these four Envs, suggesting that the relative resistance of these Envs over others tested here are probably due to changes outside the MPER. Nonetheless, these 4 Envs showed 30-40% sensitivity to 4E10 at a concentration of 6.66 μg/ml, indicating these Envs required excess 4E10 for 50% neutralization but certainly not as much as that would require for W680G or F673N as shown by Nakamura *et al *[78]. One Env variant each from subjects IVC2 and IVC 3 obtained at first follow up visits that showed unusual sensitivity to 17b, indicating exposed CD4i epitopes. These two Env variants in contrast to the majority of the Env clones were also found to be efficient at infecting HeLa cells expressing low levels of CD4 thereby indicating the presence of exposed CD4i epitopes on Env that enabled them to productively infect HeLa cells expressing low CD4. Nonetheless, two Env variants (5.4.J22 and 5.4.J49) obtained from IVC 5 patient at 2 years showed increased infectivity to HeLa cells expressing low CD4 but were resistant to 17b, indicating that these Envs evolved to conceal their coreceptor binding region on gp120 without compromising low CD4 dependence in the same way that most circulating variants do.

How NAbs drive the Env evolution that impacts on CD4 affinity, tropism and sensitivity to NAbs is not very clear in early HIV-1 clade C infection although two groups using HIV-1 clade B Envs showed association of R5 macrophage tropism with increased CD4 affinity consistent with increased resistance to anti-CD4 monoclonal antibodies [79,80]. Although in general, the majority of the Envs obtained from all the patients were moderately sensitive to sCD4, we found a few Envs (5.J41, 4-5.J5, 5-4.J16, 11.J25 and 11.J28) that showed autologous antibody resistance but were moderately sensitive to sCD4 indicating that these Envs evolved strategies in escaping autologous neutralization however they retained a very high affinity for the CD4 receptor. The CD4 binding site (CD4bs) on Env experiences most selective pressure as potent NAbs are directed to this domain as documented earlier [15,49]. Under this selective pressure exerted by humoral immunity, CD4bs is compelled to acquire changes in Env sequences to escape from NAbs that in turn would restrict Env binding efficiently to CD4 receptors [81]. In our study we found that all the Envs that were sensitive to autologous plasma antibodies were moderately susceptible to sCD4 indicating in this scenario, autologous NAbs were mostly directed towards the CD4 binding domain and escape from NAbs possibly had compromised Env binding with CD4. When tested for the extent of CD4 exposure of gp120, Envs that were sensitive to autologous antibodies as well as to sCD4 were found to require less cell surface CD4 for efficient entry, indicating an inverse correlation between Env sensitivity to autologous NAbs and CD4 dependence. The relationship between sensitivity of Envs to sCD4 and anti-CD4 antibodies with variable dependence to cell surface CD4 were described previously by different investigators. Gorry *et al *[82] showed that a neurotropic Env obtained from brain tissue with higher affinity to CD4 was found to be increasingly sensitive to CD4 mimetic, CD4-IgG2. Later, Dunfee *et al *[83] showed that Envs with N283 substitution could productively infect cells expressing low cell surface CD4 and show greater affinity to sCD4. Similar observations were found by Vermeire *et *al [81], where they showed that a NL4-3 variant that evolved to infect cells expressing low CD4 in presence of the small molecule CADA was found to be highly susceptible to heterologous sera and was concordant with increased sensitivity and resistance to sCD4 and anti-CD4 respectively. In addition, Peters *et al *[79,84] demonstrated that patient-derived Envs that were able to exploit low CD4 on cell surface were proportionately resistant and sensitive to anti-CD4 antibody and sCD4 respectively.

In conclusion, in the present study, we have shown for the first time the neutralization properties of HIV-1 India clade C Env clones obtained from patients followed up with recent infection over time to their autologous antibodies during the natural course of infection and investigated their genetic relatedness with sensitivity to known broadly neutralizing monoclonal antibodies and degree of exposure to CD4 for efficient entry. While variations in autologous neutralization of viruses are expected, all available data on the mechanisms of resistance and sensitivity to neutralizing antibodies of geographically diversified HIV-1 clade C that contributes to major global HIV-1 pandemic will help designing strategies fostering vaccine discovery.

## Methods

### Patient details, PBMC and plasma samples

All five recently infected study subjects acquired HIV-1 through heterosexual contacts and were ART naïve at the time of blood collection. The mean CD4 counts ranged from 328-606 cells per cubic millimeter (mm^3^). Based on detuned ELISA results [85-87] and history of exposure within the last 6 to 8 months, these patients were selected as recently infected patients for further characterization. Plasmas used for HIV-1 envelope amplification and tested for antibody assays were obtained at baseline, 6 and 12 months respectively.

### Amplification and cloning of gp160

*gp160 *amplification from peripheral blood mononuclear cell (PBMC) DNA and from reverse-transcribed plasma viral RNA was carried out by nested PCR using 5'-TAGAGCCCTGGAAGCATCCAGGAAG-3' as forward and 5'-TTGCTACTTGTGATTGCTCCATGT-3' as reverse primer in the first round and 5'-CACCGGCTTAGGCATCTCCTATGGCAGGAAGAA-3' as forward and 5'-TATCGGTACCAGTCTTGAGACGCTGCTCCTACTC-3' as reverse primer in the second round by using Platinum Taq proof reading polymerase (Invitrogen Inc.). Plasma viral RNA was purified by using a nucleic acid isolation kit as described by the manufacturer (Roche Inc.). cDNA from diluted viral RNA was prepared using Superscript III first strand synthesis kit (Invitrogen Inc.). gp160 was amplified by two rounds of nested PCR gp160 amplicons were purified and ligated into either pcDNA 3.1/V5-His-TOPO (Invitrogen Inc) or pSVIIIenv [84].

### DNA sequencing and phylogenetic analysis

Sequence analysis was performed using cycle sequencing and big dye terminator methods by automated genetic analyzer (Applied Biosystems, Inc; Model 3730XL) as described earlier [88]. Nucleotide and deduced amino acid sequences were aligned using MEGA software and phylogenetic tree was constructed by the neighbor-joining method [88].

### Pseudovirion preparation and measurement of virus titer

Pseudotyped viruses carrying patient Envelope were produced by cotransfection of env^+ ^pSVIIIenv or env^+ ^pcDNA 3.1/V5-His-TOPO with env-defective HIV-1 backbone vector (pSG3ΔEnv) [44,89], into 293T cells during log growth phase in 6-well tissue culture trays (Corning Inc) using calcium phosphate (Promega Inc) following manufacturer's protocol. Cell supernatants carrying progeny pseudotyped viruses were harvested at 48 hours post-transfection, and stored at -152°C until further usage. The infectivity assays were done in TZM-bl cells in 96-well microtiter plate and infectivity titers determined by measuring the luciferase activity respectively as described elsewhere [90].

### Neutralization Assay

Patient plasma samples were evaluated for NAb activity against Env pseudotyped viruses using a single round reporter assay in TZM-bl cells as described previously with few modification [90]. Briefly, 200 TCID_50 _of pseudovirus was incubated with serial 3 fold dilutions of plasma sample in duplicates in a total volume of 150 μl for 1 hr at 37°C in 96-well flat-bottom culture plates. Freshly trypsinized cells (10,000 cells in 100 μl of growth medium containing 25 ug/ml DEAE Dextran) were added to each well. One set of control wells received cells plus pseudovirus (virus control) and another set received cells only (background control). After 48 hours of incubation, luciferase activity was measured by using the Bright-Glo Luciferase Assay System (Promega Inc.). The 50% inhibitory dose (ID_50_) was defined as either the plasma dilution or sample concentration (in the case of sCD4 and MAbs) that caused a 50% reduction in relative luminescence units (RLU) compared to virus control wells after subtraction of background RLU.

### p24 antigen immunostaining

Immunostaining of HeLa cells infected with pseudoviruses was carried out as described earlier [84]. p24 positive cells were regarded as foci of infection, and virus infectivity was estimated as focus-forming units (FFU) per milliliter.

### Nucleotide sequence accession numbers

All env sequences have been submitted to GenBank (accession numbers: [GenBank:EU908214] to [GenBank:EU908221], [GenBank:EU908224] to [GenBank:EU908225], and [GenBank:GU945306] to [GenBank:GU945333]).

### Statistical analyses

Correlations between NAb response and magnitude of envelope binding to sCD4, RC49 cells and anti-CD4 antibody (SIM.2) were assessed by calculating Spearman's non-parametric 2-tailed correlation co-efficient with 95% confidence level using GraphPad Prism software. The percent infectivity of Env clones in HeLa cells expressing low CD4 (RC49) were plotted and compared by Mann-Whitney and two-way ANNOVA tests using GraphPad Prism software. Correlations were considered significant with P values less than 0.05. To avoid digression of correlation, one Env clone (4.2J45) was not included during assessing the correlation between Env sensitivity to NAb and sCD4 (Figure 5) and between NAb and HeLa cell (RC49) (Figure 6B) infection as the sensitivity of this Env clone to it autologous plasma was exceptionally higher (ID_50 _greater than 6000; see Table 3)

## Abbreviations

Env: (envelope); NAb: (neutralizing antibody); sCD4: (soluble CD4); MAb: (monoclonal antibody)

## Competing interests

The authors declare that they have no competing interests.

## Authors' contributions

JB conceptualized and planned the study; RR carried out molecular cloning, neutralization assays and majority of the experiments; MT recruited patients with recent infections, did detuned ELISA and provided essential patient information including CD4 counts; RR and JB analyzed sequence analyses; JB wrote the manuscript with the help of RR and MT. All the authors have read and approved the final manuscript.

## Supplementary Material

Additional file 1 **Figure S1**. Alignments of deduced amino acids of Indian clade C patient envelopes obtained at different course of infection. Nucleotide sequences were translated and aligned using Mega 4.1. The residues were started from KpnI site in gp120 and did not include signal peptide. While dashes denote sequence identity in Env, dots indicate gaps. Letters in lowercase in the consensus sequence indicate residues under represented at that position in Envs obtained from all the patients. Residues that differed significantly at a particular position were denoted as X in the consensus sequence. Potential N-linked glycosylation sites were underscored and highlighted. Click here for file

Additional file 2 **Table S1**. 2F5 and 4E10 minimum motifs in MPER domain in patient Envs and their corresponding sensitivities to 2F5 and 4E10 monoclonal antibodies. Click here for file

Additional file 3 **Figure S2**. Variations in CD4 dependence of patient Envs obtained at different time points in each patient. Note that the bar represents the median percentage infectivity of pseudoviruses to RC49 cells expressing low CD receptors. Click here for file

## References

[B1] Haynes BF Montefiori DC Aiming to induce broadly reactive neutralizing antibody responses with HIV-1 vaccine candidates Expert Rev Vaccines 2006 5 347 363 10.1586/14760584.5.3.347 16827619 PMC2716009

[B2] Hu SL Stamatatos L Prospects of HIV Env modification as an approach to HIV vaccine design Curr HIV Res 2007 5 507 513 10.2174/157016207782418542 18045108

[B3] Phogat S Wyatt R Rational modifications of HIV-1 envelope glycoproteins for immunogen design Curr Pharm Des 2007 13 213 227 10.2174/138161207779313632 17269929

[B4] Burton DR Desrosiers RC Doms RW Koff WC Kwong PD Moore JP Nabel GJ Sodroski J Wilson IA Wyatt RT HIV vaccine design and the neutralizing antibody problem Nat Immunol 2004 5 233 236 10.1038/ni0304-233 14985706

[B5] Karlsson Hedestam GB Fouchier RA Phogat S Burton DR Sodroski J Wyatt RT The challenges of eliciting neutralizing antibodies to HIV-1 and to influenza virus Nat Rev Microbiol 2008 6 143 155 10.1038/nrmicro1819 18197170

[B6] Zhou T Xu L Dey B Hessell AJ Van Ryk D Xiang SH Yang X Zhang MY Zwick MB Arthos J Burton DR Dimitrov DS Sodroski J Wyatt R Nabel GJ Kwong PD Structural definition of a conserved neutralization epitope on HIV-1 gp120 Nature 2007 445 732 737 10.1038/nature05580 17301785 PMC2584968

[B7] Braibant M Brunet S Costagliola D Rouzioux C Agut H Katinger H Autran B Barin F Antibodies to conserved epitopes of the HIV-1 envelope in sera from long-term non-progressors: prevalence and association with neutralizing activity Aids 2006 20 1923 1930 10.1097/01.aids.0000247113.43714.5e 16988513

[B8] Donners H Willems B Beirnaert E Colebunders R Davis D van der Groen G Cross-neutralizing antibodies against primary isolates in African women infected with HIV-1 Aids 2002 16 501 503 10.1097/00002030-200202150-00030 11834970

[B9] Gray ES Moore PL Choge IA Decker JM Bibollet-Ruche F Li H Leseka N Treurnicht F Mlisana K Shaw GM Karim SS Williamson C Morris L CAPRISA 002 Study Team Neutralizing antibody responses in acute human immunodeficiency virus type 1 subtype C infection J Virol 2007 81 6187 6196 10.1128/JVI.00239-07 17409164 PMC1900112

[B10] Li B Decker JM Johnson RW Bibollet-Ruche F Wei X Mulenga J Allen S Hunter E Hahn BH Shaw GM Blackwell JL Derdeyn CA Evidence for potent autologous neutralizing antibody titers and compact envelopes in early infection with subtype C human immunodeficiency virus type 1 J Virol 2006 80 5211 5218 10.1128/JVI.00201-06 16699001 PMC1472127

[B11] Moog C Fleury HJ Pellegrin I Kirn A Aubertin AM Autologous and heterologous neutralizing antibody responses following initial seroconversion in human immunodeficiency virus type 1-infected individuals J Virol 1997 71 3734 3741 9094648 10.1128/jvi.71.5.3734-3741.1997 PMC191523

[B12] Pilgrim AK Pantaleo G Cohen OJ Fink LM Zhou JY Zhou JT Bolognesi DP Fauci AS Montefiori DC Neutralizing antibody responses to human immunodeficiency virus type 1 in primary infection and long-term-nonprogressive infection J Infect Dis 1997 176 924 932 10.1086/516508 9333150

[B13] Richman DD Wrin T Little SJ Petropoulos CJ Rapid evolution of the neutralizing antibody response to HIV type 1 infection Proc Natl Acad Sci USA 2003 100 4144 4149 10.1073/pnas.0630530100 12644702 PMC153062

[B14] Kraft Z Strouss K Sutton WF Cleveland B Tso FY Polacino P Overbaugh J Hu SL Stamatatos L Characterization of neutralizing antibody responses elicited by clade A envelope immunogens derived from early transmitted viruses J Virol 2008 82 5912 5921 10.1128/JVI.00389-08 18400850 PMC2395128

[B15] Li Y Migueles SA Welcher B Svehla K Phogat A Louder MK Wu X Shaw GM Connors M Wyatt RT Mascola JR Broad HIV-1 neutralization mediated by CD4-binding site antibodies Nat Med 2007 13 1032 1034 10.1038/nm1624 17721546 PMC2584972

[B16] Wyatt R Sodroski J The HIV-1 envelope glycoproteins: fusogens, antigens, and immunogens Science 1998 280 1884 1888 10.1126/science.280.5371.1884 9632381

[B17] Parren PW Mondor I Naniche D Ditzel HJ Klasse PJ Burton DR Sattentau QJ Neutralization of human immunodeficiency virus type 1 by antibody to gp120 is determined primarily by occupancy of sites on the virion irrespective of epitope specificity J Virol 1998 72 3512 3519 9557629 10.1128/jvi.72.5.3512-3519.1998 PMC109569

[B18] Ugolini S Mondor I Parren PW Burton DR Tilley SA Klasse PJ Sattentau QJ Inhibition of virus attachment to CD4+ target cells is a major mechanism of T cell line-adapted HIV-1 neutralization J Exp Med 1997 186 1287 1298 10.1084/jem.186.8.1287 9334368 PMC2199094

[B19] Labrijn AF Poignard P Raja A Zwick MB Delgado K Franti M Binley J Vivona V Grundner C Huang CC Venturi M Petropoulos CJ Wrin T Dimitrov DS Robinson J Kwong PD Wyatt RT Sodroski J Burton DR Access of antibody molecules to the conserved coreceptor binding site on glycoprotein gp120 is sterically restricted on primary human immunodeficiency virus type 1 J Virol 2003 77 10557 10565 10.1128/JVI.77.19.10557-10565.2003 12970440 PMC228502

[B20] Deeks SG Schweighardt B Wrin T Galovich J Hoh R Sinclair E Hunt P McCune JM Martin JN Petropoulos CJ Hecht FM Neutralizing antibody responses against autologous and heterologous viruses in acute versus chronic human immunodeficiency virus (HIV) infection: evidence for a constraint on the ability of HIV to completely evade neutralizing antibody responses J Virol 2006 80 6155 6164 10.1128/JVI.00093-06 16731954 PMC1472617

[B21] Doria-Rose NA Klein RM Manion MM O'Dell S Phogat A Chakrabarti B Hallahan CW Migueles SA Wrammert J Ahmed R Nason M Wyatt RT Mascola JR Connors M Frequency and phenotype of human immunodeficiency virus envelope-specific B cells from patients with broadly cross-neutralizing antibodies J Virol 2009 83 188 199 10.1128/JVI.01583-08 18922865 PMC2612342

[B22] Binley JM Lybarger EA Crooks ET Seaman MS Gray E Davis KL Decker JM Wycuff D Harris L Hawkins N Wood B Nathe C Richman D Tomaras GD Bibollet-Ruche F Robinson JE Morris L Shaw GM Montefiori DC Mascola JR Profiling the specificity of neutralizing antibodies in a large panel of plasmas from patients chronically infected with human immunodeficiency virus type 1 subtypes B and C J Virol 2008 82 11651 11668 10.1128/JVI.01762-08 18815292 PMC2583680

[B23] Simek MD Rida W Priddy FH Pung P Carrow E Laufer DS Lehrman JK Boaz M Tarragona-Fiol T Miiro G Birungi J Pozniak A McPhee DA Manigart O Karita E Inwoley A Jaoko W Dehovitz J Bekker LG Pitisuttithum P Paris R Walker LM Poignard P Wrin T Fast PE Burton DR Koff WC Human immunodeficiency virus type 1 elite neutralizers: individuals with broad and potent neutralizing activity identified by using a high-throughput neutralization assay together with an analytical selection algorithm J Virol 2009 83 7337 7348 10.1128/JVI.00110-09 19439467 PMC2704778

[B24] Mascola JR Montefiori DC The role of antibodies in HIV vaccines Annu Rev Immunol 2010 28 413 444 10.1146/annurev-immunol-030409-101256 20192810

[B25] Burton DR Pyati J Koduri R Sharp SJ Thornton GB Parren PW Sawyer LS Hendry RM Dunlop N Nara PL Efficient neutralization of primary isolates of HIV- 1 by a recombinant human monoclonal antibody Science 1994 266 1024 1027 10.1126/science.7973652 7973652

[B26] Binley JM Wrin T Korber B Zwick MB Wang M Chappey C Stiegler G Kunert R Zolla-Pazner S Katinger H Petropoulos CJ Burton DR Comprehensive cross-clade neutralization analysis of a panel of anti-human immunodeficiency virus type 1 monoclonal antibodies J Virol 2004 78 13232 13252 10.1128/JVI.78.23.13232-13252.2004 15542675 PMC524984

[B27] Kulkarni SS Lapedes A Tang H Gnanakaran S Daniels MG Zhang M Bhattacharya T Li M Polonis VR McCutchan FE Morris L Ellenberger D Butera ST Bollinger RC Korber BT Paranjape RS Montefiori DC Highly complex neutralization determinants on a monophyletic lineage of newly transmitted subtype C HIV-1 Env clones from India Virology 2009 385 505 520 10.1016/j.virol.2008.12.032 19167740 PMC2677301

[B28] Trkola A Purtscher M Muster T Ballaun C Buchacher A Sullivan N Srinivasan K Sodroski J Moore JP Katinger H Human monoclonal antibody 2G12 defines a distinctive neutralization epitope on the gp120 glycoprotein of human immunodeficiency virus type 1 J Virol 1996 70 1100 1108 8551569 10.1128/jvi.70.2.1100-1108.1996 PMC189917

[B29] Calarese DA Scanlan CN Zwick MB Deechongkit S Mimura Y Kunert R Zhu P Wormald MR Stanfield RL Roux KH Kelly JW Rudd PM Dwek RA Katinger H Burton DR Wilson IA Antibody domain exchange is an immunological solution to carbohydrate cluster recognition Science 2003 300 2065 2071 10.1126/science.1083182 12829775

[B30] Trkola A Pomales AB Yuan H Korber B Maddon PJ Allaway GP Katinger H Barbas CF Burton DR Ho DD Cross-clade neutralization of primary isolates of human immunodeficiency virus type 1 by human monoclonal antibodies and tetrameric CD4-IgG J Virol 1995 69 6609 6617 7474069 10.1128/jvi.69.11.6609-6617.1995 PMC189569

[B31] Li M Salazar-Gonzalez JF Derdeyn CA Morris L Williamson C Robinson JE Decker JM Li Y Salazar MG Polonis VR Mlisana K Karim SA Hong K Greene KM Bilska M Zhou J Allen S Chomba E Mulenga J Vwalika C Gao F Zhang M Korber BT Hunter E Hahn BH Montefiori DC Genetic and neutralization properties of subtype C human immunodeficiency virus type 1 molecular env clones from acute and early heterosexually acquired infections in Southern Africa J Virol 2006 80 11776 11790 10.1128/JVI.01730-06 16971434 PMC1642599

[B32] Decker JM Bibollet-Ruche F Wei X Wang S Levy DN Wang W Delaporte E Peeters M Derdeyn CA Allen S Hunter E Saag MS Hoxie JA Hahn BH Kwong PD Robinson JE Shaw GM Antigenic conservation and immunogenicity of the HIV coreceptor binding site J Exp Med 2005 201 1407 1419 10.1084/jem.20042510 15867093 PMC2213183

[B33] Wyatt R Kwong PD Desjardins E Sweet RW Robinson J Hendrickson WA Sodroski JG The antigenic structure of the HIV gp120 envelope glycoprotein Nature 1998 393 705 711 10.1038/31514 9641684

[B34] Thali M Moore JP Furman C Charles M Ho DD Robinson J Sodroski J Characterization of conserved human immunodeficiency virus type 1 gp120 neutralization epitopes exposed upon gp120-CD4 binding J Virol 1993 67 3978 3988 7685405 10.1128/jvi.67.7.3978-3988.1993 PMC237765

[B35] Xiang SH Doka N Choudhary RK Sodroski J Robinson JE Characterization of CD4-induced epitopes on the HIV type 1 gp120 envelope glycoprotein recognized by neutralizing human monoclonal antibodies AIDS Res Hum Retroviruses 2002 18 1207 1217 10.1089/08892220260387959 12487827

[B36] Moulard M Phogat SK Shu Y Labrijn AF Xiao X Binley JM Zhang MY Sidorov IA Broder CC Robinson J Parren PW Burton DR Dimitrov DS Broadly cross-reactive HIV-1-neutralizing human monoclonal Fab selected for binding to gp120-CD4-CCR5 complexes Proc Natl Acad Sci USA 2002 99 6913 6918 10.1073/pnas.102562599 11997472 PMC124503

[B37] Cardoso RM Zwick MB Stanfield RL Kunert R Binley JM Katinger H Burton DR Wilson IA Broadly neutralizing anti-HIV antibody 4E10 recognizes a helical conformation of a highly conserved fusion-associated motif in gp41 Immunity 2005 22 163 173 10.1016/j.immuni.2004.12.011 15723805

[B38] Zwick MB Labrijn AF Wang M Spenlehauer C Saphire EO Binley JM Moore JP Stiegler G Katinger H Burton DR Parren PW Broadly neutralizing antibodies targeted to the membrane-proximal external region of human immunodeficiency virus type 1 glycoprotein gp41 J Virol 2001 75 10892 10905 10.1128/JVI.75.22.10892-10905.2001 11602729 PMC114669

[B39] Muster T Steindl F Purtscher M Trkola A Klima A Himmler G Ruker F Katinger H A conserved neutralizing epitope on gp41 of human immunodeficiency virus type J Virol 1993 67 6642 6647 7692082 10.1128/jvi.67.11.6642-6647.1993 PMC238102

[B40] Binley JM Cayanan CS Wiley C Schulke N Olson WC Burton DR Redox-triggered infection by disulfide-shackled human immunodeficiency virus type 1 pseudovirions J Virol 2003 77 5678 5684 10.1128/JVI.77.10.5678-5684.2003 12719560 PMC154040

[B41] Yuste E Sanford HB Carmody J Bixby J Little S Zwick MB Greenough T Burton DR Richman DD Desrosiers RC Johnson WE Simian immunodeficiency virus engrafted with human immunodeficiency virus type 1 (HIV-1)-specific epitopes: replication, neutralization, and survey of HIV-1-positive plasma J Virol 2006 80 3030 3041 10.1128/JVI.80.6.3030-3041.2006 16501112 PMC1395451

[B42] Gray ES Taylor N Wycuff D Moore PL Tomaras GD Wibmer CK Puren A DeCamp A Gilbert PB Wood B Montefiori DC Binley JM Shaw GM Haynes BF Mascola JR Morris L Antibody specificities associated with neutralization breadth in plasma from human immunodeficiency virus type 1 subtype C-infected blood donors J Virol 2009 83 8925 8937 10.1128/JVI.00758-09 19553335 PMC2738176

[B43] Albert J Abrahamsson B Nagy K Aurelius E Gaines H Nystrom G Fenyo EM Rapid development of isolate-specific neutralizing antibodies after primary HIV-1 infection and consequent emergence of virus variants which resist neutralization by autologous sera Aids 1990 4 107 112 10.1097/00002030-199002000-00002 2328092

[B44] Wei X Decker JM Wang S Hui H Kappes JC Wu X Salazar-Gonzalez JF Salazar MG Kilby JM Saag MS Komarova NL Nowak MA Hahn BH Kwong PD Shaw GM Antibody neutralization and escape by HIV-1 Nature 2003 422 307 312 10.1038/nature01470 12646921

[B45] Bunnik EM Pisas L van Nuenen AC Schuitemaker H Autologous neutralizing humoral immunity and evolution of the viral envelope in the course of subtype B human immunodeficiency virus type 1 infection J Virol 2008 82 7932 7941 10.1128/JVI.00757-08 18524815 PMC2519599

[B46] Moore PL Gray ES Choge IA Ranchobe N Mlisana K Abdool Karim SS Williamson C Morris L The c3-v4 region is a major target of autologous neutralizing antibodies in human immunodeficiency virus type 1 subtype C infection J Virol 2008 82 1860 1869 10.1128/JVI.02187-07 18057243 PMC2258729

[B47] Davis KL Gray ES Moore PL Decker JM Salomon A Montefiori DC Graham BS Keefer MC Pinter A Morris L Hahn BH Shaw GM High titer HIV-1 V3-specific antibodies with broad reactivity but low neutralizing potency in acute infection and following vaccination Virology 2009 387 414 26 10.1016/j.virol.2009.02.022 19298995 PMC2792036

[B48] Salazar-Gonzalez JF Salazar MG Keele BF Learn GH Giorgi EE Li H Decker JM Wang S Baalwa J Kraus MH Parrish NF Shaw KS Guffey MB Bar KJ Davis KL Ochsenbauer-Jambor C Kappes JC Saag MS Cohen MS Mulenga J Derdeyn CA Allen S Hunter E Markowitz M Hraber P Perelson AS Bhattacharya T Haynes BF Korber BT Hahn BH Shaw GM Genetic identity, biological phenotype, and evolutionary pathways of transmitted/founder viruses in acute and early HIV-1 infection J Exp Med 2009 206 1273 1289 10.1084/jem.20090378 19487424 PMC2715054

[B49] Rong R Bibollet-Ruche F Mulenga J Allen S Blackwell JL Derdeyn CA Role of V1V2 and other human immunodeficiency virus type 1 envelope domains in resistance to autologous neutralization during clade C infection J Virol 2007 81 1350 1359 10.1128/JVI.01839-06 17079307 PMC1797511

[B50] Rong R Gnanakaran S Decker JM Bibollet-Ruche F Taylor J Sfakianos JN Mokili JL Muldoon M Mulenga J Allen S Hahn BH Shaw GM Blackwell JL Korber BT Hunter E Derdeyn CA Unique mutational patterns in the envelope alpha 2 amphipathic helix and acquisition of length in gp120 hypervariable domains are associated with resistance to autologous neutralization of subtype C human immunodeficiency virus type 1 J Virol 2007 81 5658 5668 10.1128/JVI.00257-07 17360739 PMC1900276

[B51] Moore PL Ranchobe N Lambson BE Gray ES Cave E Abrahams MR Bandawe G Mlisana K Abdool Karim SS Williamson C Morris L Limited neutralizing antibody specificities drive neutralization escape in early HIV-1 subtype C infection PLoS Pathog 2009 5 e1000598 10.1371/journal.ppat.1000598 19763271 PMC2742164

[B52] Rong R Li B Lynch RM Haaland RE Murphy MK Mulenga J Allen SA Pinter A Shaw GM Hunter E Robinson JE Gnanakaran S Derdeyn CA Escape from autologous neutralizing antibodies in acute/early subtype C HIV-1 infection requires multiple pathways PLoS Pathog 2009 5 e1000594 10.1371/journal.ppat.1000594 19763269 PMC2741593

[B53] Bouma P Leavitt M Zhang PF Sidorov IA Dimitrov DS Quinnan GV Jr Multiple interactions across the surface of the gp120 core structure determine the global neutralization resistance phenotype of human immunodeficiency virus type 1 J Virol 2003 77 8061 8071 10.1128/JVI.77.14.8061-8071.2003 12829845 PMC161940

[B54] Carrillo A Ratner L Cooperative effects of the human immunodeficiency virus type 1 envelope variable loops V1 and V3 in mediating infectivity for T cells J Virol 1996 70 1310 1316 8551601 10.1128/jvi.70.2.1310-1316.1996 PMC189949

[B55] Cheng-Mayer C Brown A Harouse J Luciw PA Mayer AJ Selection for neutralization resistance of the simian/human immunodeficiency virus SHIVSF33A variant in vivo by virtue of sequence changes in the extracellular envelope glycoprotein that modify N-linked glycosylation J Virol 1999 73 5294 5300 10364275 10.1128/jvi.73.7.5294-5300.1999 PMC112584

[B56] Gram GJ Hemming A Bolmstedt A Jansson B Olofsson S Akerblom L Nielsen JO Hansen JE Identification of an N-linked glycan in the V1-loop of HIV-1 gp120 influencing neutralization by anti-V3 antibodies and soluble CD4 Arch Virol 1994 139 253 261 10.1007/BF01310789 7832633

[B57] Koito A Harrowe G Levy JA Cheng-Mayer C Functional role of the V1/V2 region of human immunodeficiency virus type 1 envelope glycoprotein gp120 in infection of primary macrophages and soluble CD4 neutralization J Virol 1994 68 2253 2259 8139010 10.1128/jvi.68.4.2253-2259.1994 PMC236701

[B58] Kolchinsky P Kiprilov E Bartley P Rubinstein R Sodroski J Loss of a single N-linked glycan allows CD4-independent human immunodeficiency virus type 1 infection by altering the position of the gp120 V1/V2 variable loops J Virol 2001 75 3435 3443 10.1128/JVI.75.7.3435-3443.2001 11238869 PMC114136

[B59] Korber B Gaschen B Yusim K Thakallapally R Kesmir C Detours V Evolutionary and immunological implications of contemporary HIV-1 variation Br Med Bull 2001 58 19 42 10.1093/bmb/58.1.19 11714622

[B60] Morikita T Maeda Y Fujii S Matsushita S Obaru K Takatsuki K The V1/V2 region of human immunodeficiency virus type 1 modulates the sensitivity to neutralization by soluble CD4 and cellular tropism AIDS Res Hum Retroviruses 1997 13 1291 1299 10.1089/aid.1997.13.1291 9339846

[B61] Pincus SH Messer KG Nara PL Blattner WA Colclough G Reitz M Temporal analysis of the antibody response to HIV envelope protein in HIV-infected laboratory workers J Clin Invest 1994 93 2505 2513 10.1172/JCI117260 7515393 PMC294468

[B62] Edwards TG Hoffman TL Baribaud F Wyss S LaBranche CC Romano J Adkinson J Sharron M Hoxie JA Doms RW Relationships between CD4 independence, neutralization sensitivity, and exposure of a CD4-induced epitope in a human immunodeficiency virus type 1 envelope protein J Virol 2001 75 5230 5239 10.1128/JVI.75.11.5230-5239.2001 11333905 PMC114929

[B63] Dumonceaux J Goujon C Joliot V Briand P Hazan U Determination of essential amino acids involved in the CD4-independent tropism of the X4 human immunodeficiency virus type 1 m7NDK isolate: role of potential N glycosylations in the C2 and V3 regions of gp120 J Virol 2001 75 5425 5428 10.1128/JVI.75.11.5425-5428.2001 11333929 PMC114953

[B64] Joliot V Goujon C Dumonceaux J Renard A Briand P Hazan U A human immunodeficiency virus Env inducible transcription system to examine consequences of gp120 expression J Virol Methods 2001 98 145 151 10.1016/S0166-0934(01)00373-1 11576641

[B65] Rossi F Querido B Nimmagadda M Cocklin S Navas-Martin S Martin-Garcia J The V1-V3 region of a brain-derived HIV-1 envelope glycoprotein determines macrophage tropism, low CD4 dependence, increased fusogenicity and altered sensitivity to entry inhibitors Retrovirology 2008 5 89 10.1186/1742-4690-5-89 18837996 PMC2576352

[B66] Esparza J Bhamarapravati N Accelerating the development and future availability of HIV-1 vaccines: why, when, where, and how? Lancet 2000 355 2061 2066 10.1016/S0140-6736(00)02360-6 10885368

[B67] Moore JP Parren PW Burton DR Genetic subtypes, humoral immunity, and human immunodeficiency virus type 1 vaccine development J Virol 2001 75 5721 5729 10.1128/JVI.75.13.5721-5729.2001 11390574 PMC114288

[B68] Osmanov S Pattou C Walker N Schwardlander B Esparza J Estimated global distribution and regional spread of HIV-1 genetic subtypes in the year 2000 J Acquir Immune Defic Syndr 2002 29 184 190 11832690 10.1097/00042560-200202010-00013

[B69] Shankarappa R Chatterjee R Learn GH Neogi D Ding M Roy P Ghosh A Kingsley L Harrison L Mullins JI Gupta P Human immunodeficiency virus type 1 env sequences from Calcutta in eastern India: identification of features that distinguish subtype C sequences in India from other subtype C sequences J Virol 2001 75 10479 10487 75 10.1128/JVI.75.21.10479-10487.2001 11581417 PMC114623

[B70] Peeters M Kuiken FB, Hahn CB, Marx P, McCutchan F, Mellors J, Mullins J, Sodroski J, Wolinksy S, Korber B Recombinant HIV sequences: their role in the global epidemic HIV sequence compendium 2000 Theoretical Biology and Biophysics Group 2000 Los Alamos National Laboratory, Los Alamos, N Mex 39 54

[B71] Gray ES Moore PL Bibollet-Ruche F Li H Decker JM Meyers T Shaw GM Morris L 4E10-resistant variants in a human immunodeficiency virus type 1 subtype C-infected individual with an anti-membrane-proximal external region-neutralizing antibody response J Virol 2008 82 2367 2375 10.1128/JVI.02161-07 18094155 PMC2258954

[B72] Gray ES Moore PL Pantophlet RA Morris L N-linked glycan modifications in gp120 of human immunodeficiency virus type 1 subtype C render partial sensitivity to 2G12 antibody neutralization J Virol 2007 81 10769 10776 10.1128/JVI.01106-07 17634239 PMC2045459

[B73] Agnihotri KD Tripathy SP Jere AP Kale SM Paranjape RS Molecular analysis of gp41 sequences of HIV type 1 subtype C from India J Acquir Immune Defic Syndr 2006 41 345 351 10.1097/01.qai.0000209898.67007.1a 16540936

[B74] Jere A Tripathy S Agnihotri K Jadhav S Paranjape R Genetic analysis of Indian HIV-1 nef: subtyping, variability and implications Microbes Infect 2004 6 279 289 10.1016/j.micinf.2003.11.012 15026015

[B75] Kurle S Tripathy S Jadhav S Agnihotri K Paranjape R Full-length gag sequences of HIV type 1 subtype C recent seroconverters from Pune, India AIDS Res Hum Retroviruses 2004 20 1113 1118 10.1089/aid.2004.20.1113 15585103

[B76] Agnihorti K Tripathy S Jere A Jadhav S Kurle S Paranjape R gp120 sequences from HIV type 1 subtype C early seroconverters in India AIDS Res HumRetroviruses 2004 20 889 894 10.1089/0889222041725217 15320993

[B77] Khan IF Vajpayee M Prasad VV Seth P Genetic diversity of HIV type 1 subtype C env gene sequences from India AIDS Res Hum Retroviruses 2007 23 934 940 10.1089/aid.2007.0036 17678478

[B78] Nakamura K Gach JS Jones L Semrau K Walter J Bibollet-Ruche F Decker JM Heath L Decker WD Sinkala M Kankasa C Thea D Mullins J Kuhn L Zwick MB Aldrovand GM 4E10-Resistant HIV-1 Isolated from Four Subjects with Rare Membrane-Proximal External Region Polymorphisms PLoS One 2010 5 3 e9786 10.1371/journal.pone.0009786 20352106 PMC2843716

[B79] Peters PJ Duenas-Decamp MJ Sullivan WM Brown R Ankghuambom C Luzuriaga K Robinson J Burton DR Bell J Simmonds P Ball J Clapham PR Variation in HIV-1 R5 macrophage-tropism correlates with sensitivity to reagents that block envelope: CD4 interactions but not with sensitivity to other entry inhibitors Retrovirology 2008 5 5 18205925 10.1186/1742-4690-5-5 PMC2268948

[B80] Dunfee RL Thomas ER Gabuzda D Enhanced macrophage tropism of HIV in brain and lymphoid tissues is associated with sensitivity to the broadly neutralizing CD4 binding site antibody b12 Retrovirology 2009 6 69 10.1186/1742-4690-6-69 19619305 PMC2717910

[B81] Vermeire K Van Laethem K Janssens W Bell TW Schols D Human immunodeficiency virus type 1 escape from cyclotriazadisulfonamide-induced CD4-targeted entry inhibition is associated with increased neutralizing antibody susceptibility J Virol 2009 83 9577 9583 10.1128/JVI.00648-09 19570853 PMC2738218

[B82] Gorry PR Taylor J Holm GH Mehle A Morgan T Cayabyab M Farzan M Wang H Bell JE Kunstman K Moore JP Wolinsky SM Gabuzda D Increased CCR5 affinity and reduced CCR5/CD4 dependence of a neurovirulent primary human immunodeficiency virus type 1 isolate J Virol 2002 76 6277 6292 10.1128/JVI.76.12.6277-6292.2002 12021361 PMC136234

[B83] Dunfee RL Thomas ER Gorry PR Wang J Taylor J Kunstman K Wolinsky SM Gabuzda D The HIV Env variant N283 enhances macrophage tropism and is associated with brain infection and dementia Proc Natl Acad Sci USA 2006 103 15160 15165 10.1073/pnas.0605513103 17015824 PMC1586182

[B84] Peters PJ Bhattacharya J Hibbitts S Dittmar MT Simmons G Bell J Simmonds P Clapham PR Biological analysis of human immunodeficiency virus type 1 R5 envelopes amplified from brain and lymph node tissues of AIDS patients with neuropathology reveals two distinct tropism phenotypes and identifies envelopes in the brain that confer an enhanced tropism and fusigenicity for macrophages J Virol 2004 78 6915 6926 10.1128/JVI.78.13.6915-6926.2004 15194768 PMC421670

[B85] Parekh BS Kennedy MS Dobbs T Pau CP Byers R Green T Hu DJ Vanichseni S Young NL Choopanya K Mastro TD McDougal JS Quantitative detection of increasing HIV type 1 antibodies after seroconversion: a simple assay for detecting recent HIV infection and estimating incidence AIDS Res Hum Retroviruses 2002 18 295 307 10.1089/088922202753472874 11860677

[B86] Parekh BS Pau CP Kennedy MS Dobbs TL McDougal JS Assessment of antibody assays for identifying and distinguishing recent from long-term HIV type 1 infection AIDS Res Hum Retroviruses 2001 17 137 146 10.1089/08892220150217229 11177393

[B87] McDougal JS Pilcher CD Parekh BS Gershy-Damet G Branson BM Marsh K Wiktor SZ Surveillance for HIV-1 incidence using tests for recent infection in resource-constrained countries Aids 2005 19 Suppl 2 S25 30 10.1097/01.aids.0000172874.90133.7a 15930838

[B88] Lakhashe S Tripathy S Paranjape R Bhattacharya J Characterization of B/C recombinants of near full-length HIV type 1 from northeastern India with mosaics identical to ARE195FL but with a different ancestral origin AIDS Res Hum Retroviruses 2008 24 92 99 10.1089/aid.2007.0214 18275353

[B89] Wei X Decker JM Liu H Zhang Z Arani RB Kilby JM Saag MS Wu X Shaw GM Kappes JC Emergence of resistant human immunodeficiency virus type 1 in patients receiving fusion inhibitor (T-20) monotherapy Antimicrob Agents Chemother 2002 46 1896 1905 10.1128/AAC.46.6.1896-1905.2002 12019106 PMC127242

[B90] Li M Gao F Mascola JR Stamatatos L Polonis VR Koutsoukos M Voss G Goepfert P Gilbert P Greene KM Bilska M Kothe DL Salazar-Gonzalez JF Wei X Decker JM Hahn BH Montefiori DC Human immunodeficiency virus type 1 env clones from acute and early subtype B infections for standardized assessments of vaccine-elicited neutralizing antibodies J Virol 2005 79 10108 10125 10.1128/JVI.79.16.10108-10125.2005 16051804 PMC1182643

