# Supplementary material for: Variations in autologous neutralization and CD4 dependence of b12 resistant HIV-1 clade C env clones obtained at different time points from antiretroviral naïve Indian patients with recent infection
Source: Retrovirology. 2010 Sep 22;7:76. doi: 10.1186/1742-4690-7-76 (PMC2955667; doi:10.1186/1742-4690-7-76)
Supplement: Additional file 1 — Figure S1. Alignments of deduced amino acids of Indian clade C patient envelopes obtained at different course of infection. Nucleotide sequences were translated and aligned using Mega 4.1. The residues were started from KpnI site in gp120 and did not include signal peptide. While dashes denote sequence identity in Env, dots indicate gaps. Letters in lowercase in the consensus sequence indicate residues under represented at that position in Envs obtained from all the patients. Residues that differed significantly at a particular position were denoted as X in the consensus sequence. Potential N-linked glycosylation sites were underscored and highlighted. [file 1742-4690-7-76-S1.PDF]

KpNI

V1V2 Loop starts

```
Consensus              GVPVWreAKTTLFCASdAKaYdXEVHNVWATHACVPTdPnPQEm?LeNVTEnFNMWkNDMVdQMheDvISLWDQSLKPCVKLTPLCVtLeC?n.......
NARI-IVC2-NEM.J8       -----RE---------D--A-VP--------------D-N---LV-E----T----K----D--HE-V-------------------T-E-VS.VNITGR
NARI-IVC2_NEM.J9       -----RET--------D--A-VP--------------D-N---LV-E----T--V-K----D--HE-V-------------------T-E-VS.VNITGR
NARI-IVC2-3_NEM.J4     -----KE---------D--A-VP--------------D-N---LV-D----N----K----D--HE-V-------------------T-E-VN.VNIT.R
NARI-IVC2-3_NEM.J7     -----KE---------D--A-VP--------------D-N---LV-D----N----K----D--HE-V-------------------T-E-VNVVNVN.I
NARI-IVC2-3_NEMJ.17    -----KE---------D--A-VP--------------D-N---LV-D----N----K----D--HE-V-------------------T-E-VN.VNIT.R
NARI-IVC2-3_NEM.J18    -----KE---------D--A-VP--------------D-N---LV-D----N----K-G--D--HE-V-------------------T-E-VN.VNIT.R
NARI-IVC2-5_NEM.J3     -----KE---------D--A-VP--------------D-N---LV-E----N----K----D--HE-V-------------------T-E-VN.VNIT.R
NARI-IVC2-5_NEM.J11    -----RE---------D--A-IP--------------D-N---LV-E----N----K----D--HE-V-------E-----------T-E-VN.VNIT.R
NARI-IVC3_NEM.J16      -----RE---------E--A-ER--------------D-N---MV-E----N----N----D--HK-I-------------------I-K-SP......Y
NARI-IVC3-3_NEM.J9     -----RE---------E--A-ER--------------D-N---MV-E----N----N----D--HK-I-------------------I-T-SP......Y
NARI-IVC3-5_NEM.J25    -----RE---------E--A-ER--------------D-N---MV-E----N----N----D--HK-I-------------------I-T-SP......Y
NARI-IVC3-5_NEM.J38    -----RE---------E--A-ER--------------D-N---MV-E----N----N----D--HK-I----E--------------I-N-SP......Y
NARI-IVC4_NEM.J2       -----KD---------D--A-DT--------------D-N---ML-D----N----K----D--HE-V-------------------T-E-VN..LTD..
NARI-IVC4_NEM.J22      -----KD---------D--A-DT--------------D-N---ML-D----N----K----D--HE-V-------------------T-E-VN..LTD..
NARI-IVC4_NEM.J27      -----KD---------D--A-DT--------------D-N---ML-D----N----K----D--HE-V-------------------T-E-VN..LTD..
NARI-IVC4-2_NEM.J41    -----KD---------D--A-DT--------------D-N---ML-D----N----K----D--HE-V-------------------T-E-TD.......
NARI-IVC4-2_NEM.J45    -----KD---------D--A-DT--------------D-N---ML-D----N----K----D--HE-V-------------------T-E-TD.......
NARI-IVC4-2_NEM.J42b   -----KD---------D--A-DT--------------D-N---ML-D----N----K----D--HE-V-------------------T-E-VNVNLTD..
NARI-IVC4-2_NEM.J45b   -----KG---------D--A-DT--------------D-N---ML-D----N----K----D--HE-V-------------------T-E-TD.......
NARI-IVC4-2_NEM.J46b   -----KD---------D--A-DT--------------D-N---ML-D----N----K----D--HE-V-------------------T-E-VNVNLTD..
NARI-IVC4-2_NEM.J47b   -----KD---------D--A-DT--------------D-N---ML-D----N----K----D--HE-V-------------------T-E-TD.......
NARI-IVC4-5_NEM.J5     -----KD---------D--A-DT--------------D-N---ML-D----N----K----D--HE-V-------------------A-E-TD.......
NARI-IVC5_NEM.J41      -----RE---------D--G-DK--------------D-N---MA-E----N----K----E--QE-I-------------------V-N-TN.......
NARI-IVC5-3_NEM.J2     -----RE---------D--G-DK--------------D-N---MA-E----N----K----E--QE-I-------------------I-N-TN.......
NARI-IVC5-3_NEM.J4     -----RE---------D--G-DK--------------D-N---MA-E----N----K----E--QE-I-------------------I-N-TN.......
NARI-IVC5-3_NEM.J5     -----RE---------D--G-DK--------------D-N---MA-E----N----K----E--QE-I-------------------I-N-TN.......
NARI-IVC5-3_NEM.J9     -----RE---------D--G-DK--------------D-N---MA-E----N----K----E--QE-I-------------------I-N-TN.......
NARI-IVC5-4_NEM.J16    -----RE---------D--G-DK----------R---D-N---MA-E----N----K----E--QE-I-------------------I-N-TN.......
NARI-IVC5-4_NEM.J18    -----RE---------D--G-DK--------------D-N---MA-E----N----K----E--QE-I-------------------I-N-TN.......
NARI-IVC5-4_NEM.J22    -----RE---------D--G-DK--------------D-N---MA-E----N----K----E--QE-I-------------------I-N-TN.......
NARI-IVC5-4_NEM.J49    -----RE---------D--G-DK--------------D-N---MA-E----N----K----E--QE-I-------------------I-N-TN.......
NARI-IVC11_NEM.J25     -----RE---------D--A-DR--------------N-E---IV-E----N----E----D--HE-V-------------------T-E-KN.......
NARI-IVC11_NEM.J28     -----RE---------D--A-DR--------------N-E---IV-E----N----E----D--HE-V-------------------T-E-KN.......
NARI-IVC11-3_NEM.J3    -----RE---------D--A-DR--------------N-E---IV-E----N----E----D--HE-V-------------------T-K-KN.......
NARI-IVC11-3_NEM.J9    -----RE---------D--A-DR--------------N-E---IV-E----N----E----D--HE-V-------------------T-E-KN.......
NARI-IVC11-3_NEM.J16   -----RE---------D--A-DR--------------N-E---IV-E----N----E----D--HE-V-------------------T-E-KN.......
NARI-IVC11-5_NEM.J12   -----RE---------D--A-DR--------------N-E---IV-E----N----E----D--HE-V-------------------T-K-KN.......   100
```

```
                                                V1V2 Loop
                        <------------------------------------------------------------------->
Consensus               XXXn?tXXXXs.XXeXkNCsFNvTTEirdrKqXvyALFYkLDivplX.....XXXsXXXX.XXYrLINCNtSaiTQACPKvXFDPIPIHYCtPAGYaILK
NARI-IVC2-NEM.J8        NYTRDEFNDTS..DEMK--S--A---VRDR-QKVY----K--IVPLD.....NKHKSNSS.EY-R-----T-AV------VN---------T----A---
NARI-IVC2_NEM.J9        NYTRDEFNDTS..DEMK--S--A---VRDR-QKVY----R--IVPLD.....NKHKSNSS.EY-R-----T-AV------VN---------T----A---
NARI-IVC2-3_NEM.J4      NSTDKEFYDTS..NEMK--S--A---VRDR-QKVY----R--IVPLD.....NKHNSNSS.EY-R-----T-AV------VN---------T----A---
NARI-IVC2-3_NEM.J7      TRNDKEFCDTS..NEMK--S--A---VRDR-QKVY----R--IVPLD.....NKHNSNSS.EY-R-----T-AV------VN---------T----A---
NARI-IVC2-3_NEMJ.17     NSTDKEFYDTS..NEMK--S--A---VRDR-QKVY----R--IVPLD.....NKHNS......-R-----T-AV------VN---------T----A---
NARI-IVC2-3_NEM.J18     NSTDKEFYDTS..NEMK--S--A---ARDR-QKVY----R--IVPLD.....NKHNSNSS.EY-R-----T-AV------VN---------T----A---
NARI-IVC2-5_NEM.J3      NSTNKEPNDTS..NEMK--S--?---VRDR-QKVY----K--IVPLD.....YKYNSSNSSEY-R-----T-AV------VN---------T----A---
NARI-IVC2-5_NEM.J11     NSTNKKSKDTS..NEMK--S--A---VRDR-QKVY----R--IVPLD.....NEH.SSNSSEY-R-----T-AV------VN---------T----A---
NARI-IVC3_NEM.J16       QGTNKSDTHIEKDQQRE--S--T---IRDK-QTVH----K--LVPLN.....GTNSSS.....-I-----T-AI------VS---------A----A---
NARI-IVC3-3_NEM.J9      NNSDN.....YKGQQRE--S--T---IRDK-QTEH----K--LVPLN.....GTNSSS.....-I-----T-AI------VS---------A----A---
NARI-IVC3-5_NEM.J25     NNSDK.......GQQRV--S--T---IRDK-QTVH----K--LVPLN.....GTNSSN.....-I-----T-AI------VS---------A----A--Q
NARI-IVC3-5_NEM.J38     QGTNKND..THIDQQRK--S--T---IRDK-QTVH----K--LVPLN.....GTNSSS.....-I-----T-AI------VS---------T----A---
NARI-IVC4_NEM.J2        .SSNKTYYNES.MQEIK--T--V---IRDR-QRVQ----K--IVSLE.....KNSSK......-R-----T-AI------VT---------T----A---
NARI-IVC4_NEM.J22       .SSNRTYYNES.MQEIK--T--V---IRDR-RRVQ----K--IVSLE.....KNSSK......-R-----T-AI------VT---------T----A---
NARI-IVC4_NEM.J27       .SSNKTYYNES.MQEIK--T--V---IRDR-QRVQ----K--IVSLE.....KNSSK......-R-----T-AI------VT---------T----A---
NARI-IVC4-2_NEM.J41     .SSNQTHYNES.MQEIK--T--V---IRDR-QRVQ----K--IVSLE.....KNSST......-R-----T-AI------VT---------T----A---
NARI-IVC4-2_NEM.J45     .SSNQTQYNEVMMEEIK--T--V---IRDR-QRVQ----K--VVSLE.....KNSSK......-R-----T-AI------VT---------T----A---
NARI-IVC4-2_NEM.J42b    .SSNKTYHNES.MQEIK--T--V---IRDR-QRVQ----K--VVSLE.....KNSSK......-R-----T-AI------AT---------T----A---
NARI-IVC4-2_NEM.J45b    .SSNQTHYNES.MQEIK--T--V---IRDR-QRVQ----K--IVSLE.....KNSSK......-R-----T-AI------VT---------T----A---
NARI-IVC4-2_NEM.J46b    .SSNRTHFNES.MQEIK--T--V---IRDR-QRVQ----K--IVSLE.....KNSSQ......-R-----T-AI------VT---------T----A---
NARI-IVC4-2_NEM.J47b    .SSNQTHYNES.MQEIK--T--V---IRDR-QRVQ----K--IVSLE.....KNSST......-R-----T-AI------VT---------T----A---
NARI-IVC4-5_NEM.J5      .SRNQTHYNES.MQEIK--T--V---IRDR-QRVQ----K--IVSLE.....KNSSQ......-R-----T-AI------VT---------T----A---
NARI-IVC5_NEM.J41       VTRNITNITSSDWEERK--S--V---IKNR-KKEY----K--IVPLENENKNNSSNGTNSSGN-I-----S-TV------IN---------A----A---
NARI-IVC5-3_NEM.J2      VTRNITNITSSDWEERK--S--V---IKDR-KKEY----K--IVPLENENKN...........-I-----S-TV------IN---------A----A---
NARI-IVC5-3_NEM.J4      VTRNITNITSSDWEERK--S--V---IKDR-KKEY----K--IVPLENENKNNSSNGTNSSGN-I-----S-TV------IN---------A----A---
NARI-IVC5-3_NEM.J5      VTRNITNITSSDWEERK--S--V---IKDR-KKEY----K--IVPLENENKNNSSNGTNSSGN-I-----S-TV------IN---------A----A---
NARI-IVC5-3_NEM.J9      VTRNITNITSSDWEERK--S--V---IKDR-KKEY----K--IVPLENENKNNSSNGTNSSGN-I-----S-TV------IN---------A----A---
NARI-IVC5-4_NEM.J16     VTRNVTNSTSSDWEERK--S--V---IKNR-MKEY----K--...........LVNGTNSSGN-I-----S-TV------IN---------A----A---
NARI-IVC5-4_NEM.J18     VTRNITNSTSSDWEERK--S--V---IRDR-KKEY----K--IVPLENENKNNSSNGTNSSGN-I-----S-TV------IN---------A----A---
NARI-IVC5-4_NEM.J22     VTRNITNSTSSDWEERK--RRKV-HFIRDR-EKEY----K--...........TSNGTNSSGN-I-----S-TV------IN---------A----A---
NARI-IVC5-4_NEM.J49     VTRNITNSTSSDWEERK--S--V---IRDR-EKEY----K--IVPLEDENKNNSSNGTNSSGN-I-----S-TV------IN---------A----A---
NARI-IVC11_NEM.J25      .VSRAPNLTNS..EDLK--S--T---LRDK-DTVY----R--IVPLN.....EGNSNGSDDST-R-----T-AI------VT---------T----V---
NARI-IVC11_NEM.J28      .VSRAPNLTNS..EDLK--S--T---LRDK-DTVY----R--IVPLN.....EGNSNGSDDST-R-----T-AI------VT---------T----V---
NARI-IVC11-3_NEM.J3     .VSPTHNLTNS..EDLK--S--T---LRDK-DTVY----R--IVPLN.....EGKSNGSDDST-R-----T-AI------VT---------T----V---
NARI-IVC11-3_NEM.J9     .VS.RANLTNS..EDLK--S--T---LRDK-DTVY----R--IVPLN.....EGKSNGSDDST-R-----T-AI------VT---------T----V---
NARI-IVC11-3_NEM.J16    .VS.RANLTNS..EDLK--S--T---LRDK-DTVY----R--IVPLN.....EGKSNGIDYST-R-----T-AI------VT---------T----V---
NARI-IVC11-5_NEM.J12    .VNHTTNMTNS..EDLK--S--T---LRDK-DTVY----R--IVPLN.....KGNSNGSDNST-R-----T-AI------VT---------T----V---   200
```

```
                                                                                        2G12 site
                                                                                                          V3 Loop
Consensus              CNnXtFNGTGPCrNVsTVQCTHGIkPVVSTQLLLNGSLaeXXImIrsenltdNaKTIIVhLNqsVEIvCtRPnNNTRkSIRIGPGQtFYATGdIIGdIRQ
NARI-IVC2-NEM.J8       --NET-------H--S--------K-------------AEGE-M-RSENLTN-V-----H--QS---V-I--N----K--------T-----D---D---
NARI-IVC2_NEM.J9       --NET-------H--S--------K-------------AEGE-M-RSENLTN-V-----H-DQS---V-I--N----K--------T-----D---D---
NARI-IVC2-3_NEM.J4     --NET-------H--S--------K-------------AEGE-M-RSENLTN-V-----H--QS---V-I--N----K--------T-----D---D---
NARI-IVC2-3_NEM.J7     --NET-------H--S--------K-------------AEGE-M-RSENLTN-V-----H--QS---V-I--N----K--------T-----D---D---
NARI-IVC2-3_NEMJ.17    --NET-------H--S--------K-------------AEGE-M-RSENLTN-V-----H--QS---V-I--N----K--------T-----DV--D---
NARI-IVC2-3_NEM.J18    --NET-------H--S--------K-------------AEGE-M-RSENLTN-V-----H--QS---V-I--N----K--------T-----D---D---
NARI-IVC2-5_NEM.J3     --NET-------H--S--------K-------------AEGE-M-RSENLTN-V-----H--QS---V-I--N----K--------T-----D---D---
NARI-IVC2-5_NEM.J11    --NET-------H--S--------K-------------AEGE-M-RSENLTN-V-----H--QS---V-I--N----K--G-----T-----D---D---
NARI-IVC3_NEM.J16      --NKV-------K--S--------R-----------G-AEEE-I-RSENLTD-A-----H--ES---N-T--G----K--------T-----G---N---
NARI-IVC3-3_NEM.J9     --NKV-------R--S--------R-------------AEEE-I-SSENLTD-A-----H--ES---N-T--G----K--------T-----G---N---
NARI-IVC3-5_NEM.J25    --NKA-------K--S--------R-------------AEEE-I-RSENLTD-A-----H--ES---N-T--G----K--------T-----G---D---
NARI-IVC3-5_NEM.J38    --NKT-------K--S--------R-------------AEEE-I-SSENLTD-A-----H--ES---N-T--G----K--------T-----E---D---
NARI-IVC4_NEM.J2       --NET-------R--S--------K-------------AEKD-M-RSENLTD-V-----H--QT---V-T--N----Q--------V-----A---D---
NARI-IVC4_NEM.J22      --NET-------R--S--------K-------------AEKD-M-RSENLTD-V-----H--QT---V-T--N----Q--------V-----A---D---
NARI-IVC4_NEM.J27      --NET-------R--S--------R-------------AEKD-M-RSENLTD-V-----H--QT---V-T--N----Q--------V-----A---D---
NARI-IVC4-2_NEM.J41    --NET-------R--S--------K-------------AEKD-M-RSENLTD-A-----H--QT---V-I--N----Q--------V-----D---D---
NARI-IVC4-2_NEM.J45    --NET-------R--S--------K-------------AEKD-M-RSENLTD-A-----H--QT---V-I--N----Q--------V-----D---D---
NARI-IVC4-2_NEM.J42b   --NET-------R--S--------K-------------AEKD-M-RSENLTD-A-----H--QT---V-I--N----Q--------V-----D---D---
NARI-IVC4-2_NEM.J45b   --NET-------R--S--------K-------------AEKD-M-RSENLTD-A-----H--QT---V-I--N----Q--------V-----D---D---
NARI-IVC4-2_NEM.J46b   --NET-------R--S--------K-------------AEKD-M-RPENLTD-A-----H--QT---V-T--N----Q--------V-----D---D---
NARI-IVC4-2_NEM.J47b   --NET-------R--S--------K-------------AEKD-M-RSENLTD-A-----H--QT---V-I--N----Q--------V-----D---D---
NARI-IVC4-5_NEM.J5     --NET-------R--S--------K-------------AEKD-M-RSENLTD-A-----H--QT---V-I--N----Q--------V-----D---D---
NARI-IVC5_NEM.J41      --ETT-------R--T--------K-------------AKEK-M-RTEKWED-A-----Q--QS---T-T--D----K--------T-----D---D---
NARI-IVC5-3_NEM.J2     --ETT-------R--T--------K-------------AKEK-M-RTEKWED-A-----Q--QS---T-T--G----K--------T-----D---D---
NARI-IVC5-3_NEM.J4     --ETT-------R--T--------K-------------AKEK-M-RTEKWED-A-----Q--QS---T-T--G----K--------T-----D---D---
NARI-IVC5-3_NEM.J5     --ETT-------R--T--------K-------------AKEK-M-RTEKWED-A-----Q--QS---T-T--G----K--------T-----D---D---
NARI-IVC5-3_NEM.J9     --ETT-------R--T--------K-------------AKEK-M-RTEKWED-A-----Q--QS---T-T--G----K--------T-----D---D---
NARI-IVC5-4_NEM.J16    --ETT-------R--T--------K-------------AKEK-M-RTENWKD-T-----Q--QS---T-T--G----K--------T-----D---D---
NARI-IVC5-4_NEM.J18    --ETT-------R--T--------K-------------AKEK-I-RTEKWED-T-----Q--QS---T-T--G----K--------T-----D---N---
NARI-IVC5-4_NEM.J22    --ETT-------R--T--------K-------------AKEK-I-RTEKWED-T-----Q--QS---T-T--G----K--------T-----D---D---
NARI-IVC5-4_NEM.J49    --ETT-------R--T--------K-------------AKEK-I-RTEKWED-T-----Q--QS---T-T--G----K--------T-----D---D---
NARI-IVC11_NEM.J25     --DKK-------S--S--------K-------------SEGE-M-RSKNLTN-A-----H--KS---V-T--N----K--------T-----D---N---
NARI-IVC11_NEM.J28     --DKK-------S--S--------K-------------SEGE-M-RSKNLTN-A-----H--KS---V-T--N----R--------T-----D---N---
NARI-IVC11-3_NEM.J3    --DKK-------S--S--------K-------------SEGE-M-RSKNLTN-A-----H--KS---V-T--N----K--------T-----D---N---
NARI-IVC11-3_NEM.J9    --DKK-------S--S--------K-------------SEGE-M-RSKNLTN-A-----H--KS---V-T--N----K--------T-----D---N---
NARI-IVC11-3_NEM.J16   --DKK-------S--S--------K-------------SEGE-M-RSKNLTN-A-----H--KS---V-T--N----K--------T-----D---N---
NARI-IVC11-5_NEM.J12   --DKK-------S--S--------K-------------SEGE-M-RSKNLTN-A-----H--KS---V-T--N----K--------T-----D---D---   300
```

```
                          V3 Loop ends                      CD4Bs                       V4 Loop
Consensus                 AXC?InXtXWneTLqrVskkLAEyfpnkTIXFapsSGGDlEITtHSFNCrGEFFYCNTSXLFNstYXXngtXXXXX....nXtXITIpCrIKQiiNmWQe
NARI-IVC2-NEM.J8          -S-T-NESR-NE--QR-SKK---YFPNK--K-APS----L---T-----R---------K---GT-MSNDTKGD......LSSV---P-R---II-M--E
NARI-IVC2_NEM.J9          -S-T-NESR-NE--QR-SKK---YFPNK--K-APS----L---T-----R---------K---GT-MSNDTKGN......LSSV---P-R---II-M--E
NARI-IVC2-3_NEM.J4        -S-T-NESR-NE--QR-SKK---YFPNK--K-APS----L---T-----R---------K---GT-MSNDTKGN......LSSV---P-R---II-M--E
NARI-IVC2-3_NEM.J7        -S-T-NESR-NE--QR-SKK---YFPNK--K-APS----L---T-----R---------K---GT-MSNDTKGN......LSSV---P-R---II-M--E
NARI-IVC2-3_NEMJ.17       -S-T-NESR-NE--QR-SKK---YFPNK--K-APS----L---T-----R---------K---GT-MSNDTKGN......LSSV---P-R---II-M--E
NARI-IVC2-3_NEM.J18       -S-T-NESR-NE--QR-SKK---YFPNK--K-APS----L---T-----R---------K---GT-MSNDTKGN......LSSV---P-R---II-M--E
NARI-IVC2-5_NEM.J3        -S-T-NEAR-NE--QR-SKK---YFPNK--N-APS----L---T-----R---------K---GT-MSNTTKGN......LSSV---P-R---II-M--E
NARI-IVC2-5_NEM.J11       -S-T-NEAR-NE--QR-SKK---YFPNK--N-APS----L---T-----R---------K---GT-MSNDTKGN......LSSV---P-R---II-M--E
NARI-IVC3_NEM.J16         -H-N-SREQ-NN--KR-RDK---HFTNT--I-APS----L---T-----R---------S---KT-LSNGTEIDGAES.NSSSV---Q-K---II-M--K
NARI-IVC3-3_NEM.J9        -H-N-SREQ-NN--KR-RDK---HFN.T--I-APS----L---T-----R---------S---KT-LSNGTEIDGAES.NSSPV---Q-K---II-M--K
NARI-IVC3-5_NEM.J25       -H-N-SREQ-NN--KR-RDK---RFANK--I-APS----L---T-----R---------S---KT-LSNGTEIDGAES.NSSSV---Q-R---II-M--K
NARI-IVC3-5_NEM.J38       -H-N-SREQ-NN--KR-RDK---RFANK--I-APS----L---T-----R---------S---KT-LSNGTEIDGAES.NSSSV---Q-R---II-M--K
NARI-IVC4_NEM.J2          -Y-T-NTTA-NE--QR-SKK---HFPNK--R-APS----L---T-----R---------G---ST-MTNGTFTYKP....NGTN---P-R---II-M--E
NARI-IVC4_NEM.J22         -Y-T-NTTA-NE--QR-SKK---HFPNK--R-APS----L---T-----R---------G---ST-MTNGTFTYKP....NGTN---P-R---II-M--E
NARI-IVC4_NEM.J27         -Y-T-NTTA-NE--QR-SKK---HFPNK--R-APS----L---T-----R---------G---ST-MTNGTFTYKP....NGTN---P-R---II-M--E
NARI-IVC4-2_NEM.J41       -Y-T-NTTA-NE--QR-SKK---HFPNK--R-APS----L---T-----R---------G---ST-MTNGTFTYKL....NDTN---P-R---II-M--E
NARI-IVC4-2_NEM.J45       -Y-T-NTTA-NE--QR-SKK---HFPNK--R-APS----L---T-----R---------G---ST-MTNGTFTYKL....NDTN---P-R---IM-M--E
NARI-IVC4-2_NEM.J42b      -Y-T-NTTA-NE--QR-SKK---HFPNK--R-APS----L---T-----R---------G---ST-MTNGTFTYKL....NDTN---P-R---II-M--E
NARI-IVC4-2_NEM.J45b      -Y-T-NTTA-NE--QR-SKK---HFPNK--R-APS----L---T-----R---------G---ST-MTNGAFTYKL....NGTN---P-R---II-M--E
NARI-IVC4-2_NEM.J46b      -Y-T-NTTA-NE--QR-SKK---HFPNK--R-APS----L---T-----R---------G---ST-MTNGTFTYKL....NGTN---P-R---II-M--E
NARI-IVC4-2_NEM.J47b      -Y-T-NTTA-NE--QR-SKK---HFPNK--R-APS----L---T-----R---------G---ST-MTNGTFTYKL....NSTN---P-R---II-M--G
NARI-IVC4-5_NEM.J5        -Y-N-STTA-NE--QR-SKK---HFPNK--R-APS----L---T-----R---------G---ST-MTNGTF...L....NGT.---P-R---II-M--E
NARI-IVC5_NEM.J41         -H-K-NKTN-AN--QL-RNR---YFPNK--N-TSH----P---M-----R---------K---SS-DPKGKKIGNAT..INDT.---P-N---FV-M--E
NARI-IVC5-3_NEM.J2        -H-K-NKTN-DN--QL-RNR---YFPNK--N-TSH----P---M-----R---------K---SS-DPEGNKIGNAT..INDT.---P-N---FV-R--E
NARI-IVC5-3_NEM.J4        -H-K-NKTN-DN--QL-RNR---YFPNK--N-TSH----P---M-----R---------K---SS-DPEGNKIGNAT..INDT.---P-N---FV-R--E
NARI-IVC5-3_NEM.J5        -H-K-NKTN-DN--QL-RNR---YFPNK--N-TSH----P---M-----R---------K---SS-DPEGNKIGNAT..INDT.---P-N---FV-R--E
NARI-IVC5-3_NEM.J9        -H-K-NKTN-DN--QL-RNR---YFPNK--N-TSH----P---M-----R---------K---SS-DPEGNKIGNAT..INDT.---P-N---FV-R--E
NARI-IVC5-4_NEM.J16       -H-N-SKTN-TN--QL-RNR---YYPNK--N-TSH----P---M-----R---------K---SS-DPKGEKIDNATSNINDTI---P-S---FV-R--E
NARI-IVC5-4_NEM.J18       -H-N-SKTN-TN--QL-RNR---YSPNK--T-TSH----P---M-----R---------K---SS-DPKGEKIDNATSNINDTI---P-S---FV-R--E
NARI-IVC5-4_NEM.J22       -H-N-SKTN-TS--QL-RNR---YSPNK--T-TSH----P---M-----R---------K---SS-DPKGEKIDNATSNINDTI---P-S---FV-M--E
NARI-IVC5-4_NEM.J49       -H-N-SKTN-TS--QL-RNR---YSPNK--T-TSH----P---M-----R---------K---SS-DPKGEKIDNATSNINDTI---P-S---FV-M--E
NARI-IVC11_NEM.J25        -Y-D-NGTI-KE--LN-SKK---YFPNK--N-TSP----L---T-----G----H----G---GT-K.NGTYTH......MNSN---P-R---IT-M--E
NARI-IVC11_NEM.J28        -Y-D-SGTI-KE--LN-SKK---YFPDK--N-TSP----L---T-----G---------G---GT-K.NGTYTH......MNSN---P-R---II-M--E
NARI-IVC11-3_NEM.J3       -Y-N-NGTI-EE--LN-SKR---YFPNK--N-TSP----L---T-----G---------G---GT-K.NGTYTK......MNSN---P-R---II-M--E
NARI-IVC11-3_NEM.J9       -Y-N-NGTI-EE--LN-SKR---YFPNK--N-TSP----L---T-----G---------G---GT-K.NGTYTK......MNSN---P-R---II-M--E
NARI-IVC11-3_NEM.J16      -Y-N-NGTI-EE--LN-SKR---YFPNK--N-TSP----L---T-----G---------G---GT-K.NGTYTK......MNSN---P-R---II-M--E
NARI-IVC11-5_NEM.J12      -Y-N-NGTI-RE--LN-SKK---YFPNK--N-TSP----L---T-----G---------G---GT-K.NGTDTH......MNSS---P-R---II-M--E   400
```

```
                                                         V5 Loop                                                   gp41 starts
Consensus                VGRAMYAPPIaGnITCXSnITGllLvRDGGXnX.XXXEtFRPgGGdMRdNWRSELYKYKVVEIkPLGvAPTXAKRRvVerEKRAvglGAvflGFLGaAGS
NARI-IVC2-NEM.J8         ----------E-N---R-N-I-LL-V----PN..STN-T---E--D--N-------------K---V---P----M-ER----VGL--VFL----T---
NARI-IVC2_NEM.J9         ----------E-N---R-N---LL-V----PN..STN-T---E--D--N-------------K---V---P----M-EK----VGL--VFL----T---
NARI-IVC2-3_NEM.J4       ----------E-N---R-N---LL-V----LN..STN-T---E--D--N-------------K---V---P----V-KK----VGL--AFL----T---
NARI-IVC2-3_NEM.J7       ----------E-N---R-N---LL-V----LN..STN-T---E--D--N-------------K---V---P----V-KK----VGL--VFL----T---
NARI-IVC2-3_NEMJ.17      ----------E-N---R-N---LL-V----LN..STN-T---E--D--N-------------K---V---P----V-KK----VGL--VFL----T---
NARI-IVC2-3_NEM.J18      ----------E-N---R-N---LL-V----LN..STN-T---E--D--N---R---------K---V---P----V-KK----VGL--VFL----T---
NARI-IVC2-5_NEM.J3       ----------E-N---R-N---LL-V----PN..NTT-T---E--D--N-------------K---V---P----V-KK----VGL--VFL----T---
NARI-IVC2-5_NEM.J11      ----------E-N---R-N---LL-V----PN..NTT-T---E--D--N-------------K---V---P----V-KK----VGL--VFL----T---
NARI-IVC3_NEM.J16        ----------A-N---I-N---LL-V----IINRTQN-T---G--D--D-------------K---I---P----V-ER----VGI--MFL----A---
NARI-IVC3-3_NEM.J9       ----------A-N---I-N---LL-V----.INSTQN-T---G--D--D-------------K---I---P----V-ER----VGI--MFL----A---
NARI-IVC3-5_NEM.J25      ----------A-S---I-N---LL-V----.INNTQN-T---G--D--D-------------K---I---P----V-ER----VGI--MFL----A---
NARI-IVC3-5_NEM.J38      ----------A-N---I-N---LL-V----.INSTQN-T---G--D--D-------------K---I---P----V-EK----VGI--MFL----A---
NARI-IVC4_NEM.J2         ----------A-N---K-N---VI-V---ENENITE-T---G--N--D-------------K---V---K----V-ER----VGL--MFL----A---
NARI-IVC4_NEM.J22        ----------A-N---K-N---VI-V---ENENITE-T---G--N--D-------------K---V---K----V-ER----VGL--MFL----A---
NARI-IVC4_NEM.J27        ----------A-N---K-N---VI-V---ENENIT.-T---G--N--D-------------K---V---K----V-ER----VGL--MFL----A---
NARI-IVC4-2_NEM.J41      ----------A-N---K-N---ML-V---KNENS.T-T---G--N--D-------------K---V---K----V-ER----VGL--VFL----A---
NARI-IVC4-2_NEM.J45      ----------A-N---K-N---ML-V---KNENSTE-T---G--N--D-------------K---V---K----V-ER----VGL--VFL----A---
NARI-IVC4-2_NEM.J42b     ----------A-N---K-N---ML-V---KNENSTE-T---G--N--D-------------K---V---K----V-ER----VGL--VFL----A---
NARI-IVC4-2_NEM.J45b     ----------A-N---K-N---ML-V---KNENSTE-T---G--N--D-------------K---V---K----V-ER----VGL--VFL----A---
NARI-IVC4-2_NEM.J46b     ----------A-N---K-N---ML-V---ENDNSTE-T---G--N--D-------------K---V---K----V-ER----VGL--VFL----A---
NARI-IVC4-2_NEM.J47b     ----------A-N---K-N---ML-V---KNENSTE-T---G--N--D-------------K---V---K----V-ER----VGL--VFL----A---
NARI-IVC4-5_NEM.J5       ----------A-N---K-N---ML-V---KIENSTE-A---G--N--D-------------K---V---K----V-ER----VGL--VFL----A---
NARI-IVC5_NEM.J41        ----------A-V---Q-N---LL-T----NAN.SRT-V---G--D--DI------------R---I---T----V-ER----A.L--MLL----V---
NARI-IVC5-3_NEM.J2       ----------A-V---Q-N---LL-T----NAN.NGT-V---G--D--D-------------R---I---T----V-ER----A.L--MLL----V---
NARI-IVC5-3_NEM.J4       ----------A-V---Q-N---LL-T----NAN.NGT-V---G--D--D-------------R---I---T----V-ER----A.L--MLL----V---
NARI-IVC5-3_NEM.J5       ----------A-V---Q-N---LL-T----NAN.NGT-V---G--D--D-------------R---I---T----V-ER----A.L--MLL----V---
NARI-IVC5-3_NEM.J9       ----------A-V---Q-N---LL-T----NAN.NGT-V---G--D--D-------------R---I---T----V-ER----A.L--MLL----V---
NARI-IVC5-4_NEM.J16      ----------A-E---Q-N---LL-T----NTT.NDT-V---G--D--D-------------R---I---T----V-ER----A.L--MLL----V---
NARI-IVC5-4_NEM.J18      ----------A-E---Q-N---LL-T----NTT.NDT-V---G--D--D-------------R---I---T----V-ER----A.L--MLL----V---
NARI-IVC5-4_NEM.J22      ----------A-E---Q-N---LL-T----NTT.NDT-V---G--D--D-------------R---I---T----V-ER----A.L--MLL----V---
NARI-IVC5-4_NEM.J49      ----------A-E---Q-N---LL-T----NTT.NDT-V---G--D--D-------------R---I---T----V-ER----A.L--MLL----V---
NARI-IVC11_NEM.J25       ----------A-N---I-K---LL-V----QNVTNGT-I---G--D--D-------------K---V---E----V-GR----VGL--VFF----A---
NARI-IVC11_NEM.J28       ----------A-N---I-K---LL-V----QNVTNGT-I---G--D--D-------------K---V---E----V-GR----VGL--VFF----A---
NARI-IVC11-3_NEM.J3      ----------A-N---I-K---LL-V----Q...NGT-I---G--D--D-------------K---V---E----V-ER----VGL--VFF----A---
NARI-IVC11-3_NEM.J9      ----------A-N---I-K---LL-V----Q...NGT-I---G--D--D-------------K---V---E----V-ER----VGL--VFF----A---
NARI-IVC11-3_NEM.J16     ----------A-N---I-K---LL-V----Q...NGT-I---G--D--D-------------K---V---E----V-ER----VGL--VFF----A---
NARI-IVC11-5_NEM.J12     ----------A-N---M-K---LL-V----QNGTNGT-I---G--D--D-------------K---V---E----A-ER----VGL--VFF----A---   500
```

Consensus	TMGAASitLTVQaRqLLSGIVQQQsNLLXAIEAQQhlLQLTVWGIKQLQtRVLAiERYLkDQQlLGIWGCSGKlICTTaVPW**N**sSWS**N**ksqXeIWX**nMTW**	
NARI-IVC2-NEM.J8	------IT----A-Q---------S---K------HL------------T----I----K---L---------L----N----S----RSQDY--N**N**---	
NARI-IVC2_NEM.J9	------IT----A-Q---------S---K------HL------------T----I----K---L---------L----N----S----RSQDY--N**N**---	
NARI-IVC2-3_NEM.J4	------IT----A-Q---------S---K------HL------------T----I----K---L---------L----N----S----RSQDY--N**N**---	
NARI-IVC2-3_NEM.J7	------IT----A-Q---------S---K------HL------------T----I----K---L---------L----N----S----RSQDY--N**N**---	
NARI-IVC2-3_NEMJ.17	------IT----A-Q---------S---K------HL------------T----I----K---L---------L----N----S----RSQDY--N**N**---	
NARI-IVC2-3_NEM.J18	------IT----A-Q---------S---K------HL------------T----I----K---L---------L----N----S----RSQDY--N**N**---	
NARI-IVC2-5_NEM.J3	------IT----A-Q---------S---K------HL------------T----I----K---L---------L----N----S----RSQDY--N**N**---	
NARI-IVC2-5_NEM.J11	------IT----A-Q---------S---K------HL------------T----I----K---L---------L----N----S----RSQDY--N**N**---	
NARI-IVC3_NEM.J16	------LA----T-K---------S---R------QM------------A----V----Q---I---------R----A----A----KSQNE--NT---	
NARI-IVC3-3_NEM.J9	------MA----T-K---------S---R------QM------------A----V----Q---I---------R----A----A----KSQNE--N**N**---	
NARI-IVC3-5_NEM.J25	------MA----T-K---------S---R------QM------------A----V----Q---I---------R----A----A----KSQNE--N**N**---	
NARI-IVC3-5_NEM.J38	------MA----T-K---------S---R------QM------------A----V----Q---I---------R----A----A----KSQNE--N**N**---	
NARI-IVC4_NEM.J2	------IT----A-Q---------R---Q------HL------------T----I----K---L---------L----A----S----KTQEE--D**N**---	
NARI-IVC4_NEM.J22	------IT----A-Q---------R---Q------HL------------T----I----K---L---------L----A----S----KTQEE--D**N**---	
NARI-IVC4_NEM.J27	------IT----A-Q---------R---Q------HL------------T----I----K---L---------L----A----S----KTQEE--D**N**---	
NARI-IVC4-2_NEM.J41	------IT----A-Q---------R---Q------HL------------T----I----K---L---------L----A----S----KTQEE--NK---	
NARI-IVC4-2_NEM.J45	------IT----A-Q---------R---Q------HL------------T----I----K---L---------L----A----S----KTQEE--N**N**---	
NARI-IVC4-2_NEM.J42b	------IT----A-Q---------R---Q------HL------------T----I----K---L---------L----A----S----KTQKE--D**N**---	
NARI-IVC4-2_NEM.J45b	------IT----A-Q---------R---Q------HL------------T----I----K---L---------L----A----S----KTQEE--N**N**---	
NARI-IVC4-2_NEM.J46b	------IT----A-Q---------R---Q------HL------------T----I----K---L---------L----A----S----KTQKE--D**N**---	
NARI-IVC4-2_NEM.J47b	------IT----A-Q---------R---Q------HL------------T----I----K---L---------L----A----S----KTQKE--D**N**---	
NARI-IVC4-5_NEM.J5	------IT----A-Q---------R---Q------HL------------T----I----K---L---------L----A----S----KTQEA--D**N**---	
NARI-IVC5_NEM.J41	------MA----A-Q---------S---S------HL------------T----I----G---L---------L----N----S----KSQDD--EN---	
NARI-IVC5-3_NEM.J2	------MA----A-Q---------S---R------HL------------T----I----R---L---------L----N----S----KSQDD--EN---	
NARI-IVC5-3_NEM.J4	------MA----A-Q---------S---R------HL------------T----I----R---L---------L----N----S----KSQDD--EN---	
NARI-IVC5-3_NEM.J5	------MA----A-Q---------S---R------HL------------T----I----R---L---------L----N----S----KSQDD--EN---	
NARI-IVC5-3_NEM.J9	------MA----A-Q---------S---R------HL------------T----I----R---L---------L----N----S----KSQDD--EN---	
NARI-IVC5-4_NEM.J16	------MA----A-Q---------S---S------HL------------T----I----K---L---------L----N----S----KSQND--EN---	
NARI-IVC5-4_NEM.J18	------MA----A-Q---------S---S------HL------------T----I----K---L---------L----N----S----KSQND--EN---	
NARI-IVC5-4_NEM.J22	------MA----A-Q---------S---S------HL------------T----I----R---L---------L----N----S----KSQND--EN---	
NARI-IVC5-4_NEM.J49	------MA----A-Q---------S---S------HL------------T----I----R---L---------L----N----S----KSQND--EN---	
NARI-IVC11_NEM.J25	------IT----A-Q---------S---K------HL------------A----V----K---L---------L----A----T----KTKHE--D**N**---	
NARI-IVC11_NEM.J28	------IT----A-Q---------S---K------HL------------A----V----K---L---------L----A----T----KTKHE--D**N**---	
NARI-IVC11-3_NEM.J3	------IT----A-Q---------S---K------QL------------A----V----K---L---------L----A----T----KTKHE--D**N**---	
NARI-IVC11-3_NEM.J9	------IT----A-Q---------S---K------QL------------A----V----K---L---------L----A----T----KTKHE--D**N**---	
NARI-IVC11-3_NEM.J16	------IT----A-Q---------S---K------QL----?-------A----V----K---L---------L----A----T----KTKHE--D**N**---	
NARI-IVC11-5_NEM.J12	------IT----A-Q---------S---K------HM------------A----V----K---L---------L----A----T----KTKRE--D**N**---	600

```
                                             2F5 motif     4E10 motif
Consensus              MqWDrEisNYTntIYrLLEXSQnQQEkNEkdLLaLDsW?nLWnWFXITNWLWYIkIFIMIvGGLiGLRIIFAVlSiVNRVRQGYSPLSfQTltPNPggPD
NARI-IVC2-NEM.J8       -Q--R-VN---NE--R---D--N---K--QE--A--R-GN--N--N--------K-----I---I--------L-I------------F--LT---GG--
NARI-IVC2_NEM.J9       -Q--R-VN---NE--R---D--N---K--QE--A--R-GN--N--N--------K-----I---I--------L-I------------F--LT---GG--
NARI-IVC2-3_NEM.J4     -Q--R-VN---NE--R---D--N---K--QE--A--R-GN--N--N--------K-----I---V--------L-I------------F--LT---GG--
NARI-IVC2-3_NEM.J7     -Q--R-VN---NE--R---D--N---K--QE--A--R-GN--N--N--------K-----I---V--------L-I------------F--LT---GG--
NARI-IVC2-3_NEMJ.17    -Q--R-VN---NE--R-?-D--N---K--QE--G--R-GN--N--N--------K-----I---V--------L-I------------F--LT---GG--
NARI-IVC2-3_NEM.J18    -Q--R-VN---NE--R---D--N---K--QE--A--R-GN--N--N--------K-----I---V--------L-I------------F--LT---GG--
NARI-IVC2-5_NEM.J3     -Q--R-VN---NE--R---D--N---K--QE--A--R-GN--N--N--------K-----V---I--------L-I------------F--LT---GG--
NARI-IVC2-5_NEM.J11    -Q--R-VN---NE--R---D--N---K--QE--A--R-GN--N--N--------K-----V---I--------L-I------------F--LT---GG--
NARI-IVC3_NEM.J16      -E--R-IS---NT--S---K--N---E--KE--E--S-KN--S--D--------K-----V---I--------I-I------------L-ILT---GG--
NARI-IVC3-3_NEM.J9     -E--K-IS---NT--S---K--N---E--KE--E--K-KI--S--D--------K-----V---I--------I-I------------F--LI---GG--
NARI-IVC3-5_NEM.J25    -E--K-IS---NT--S---E--N---K--KE--E--K-KN--S--D--------K-----V---I--------I-I------------F--LI---GG-G
NARI-IVC3-5_NEM.J38    -E--K-IS---NT--S---E--N---K--KE--E--S-KN--S--D--------K-----V---I--------I-I------------F--LI---GG--
NARI-IVC4_NEM.J2       -E--K-IS---QT--R---D--N---K--QD--A--S-NN--N--G------H-K-----V---I--------L-I------------L--LI---GG--
NARI-IVC4_NEM.J22      -E--K-IS---QT--R---D--N---K--QD--A--S-NS--N--G--------K-----V---I--------L-I------------L--LI---GG--
NARI-IVC4_NEM.J27      -E--K-IS---QT--R---D--N---K--QD--A--S-NN--N--G--------K-----V---I--------L-I------------L--LI---GG--
NARI-IVC4-2_NEM.J41    -E--K-IS---QT--R---D--N---K--QD--A--S-NN--N--D--------K-----V---I--------L-I------------L--LI---GG--
NARI-IVC4-2_NEM.J45    -E--K-IS---QT--R---D--N---K--QD--A--S-NN--N--D--------K-----V---I--------L-I------------L--LI---GG--
NARI-IVC4-2_NEM.J42b   -E--K-ISK--QT--R---D--N---K--QD--A--S-NN--N--G--------K-----V---I--------L-I------------L--LI---GG--
NARI-IVC4-2_NEM.J45b   -E--K-IS---QT--R---D--N---K--QD--A--S-NN--N--D--------K-----V---I--------L-I------------L--LI---GG--
NARI-IVC4-2_NEM.J46b   -E--K-IS---QT--R---D--N---K--QD--A--S-NN--N--G--------K-----V---I--------L-I------------L--LI---GG--
NARI-IVC4-2_NEM.J47b   -E--K-IS---QT--R---D--N---K--QD--A--S-NN--N--G--------K-----V---I--------L-I------------L--LI---GG--
NARI-IVC4-5_NEM.J5     -E--K-VS---QT--R---D--N---K--QD--A--S-NN--N--D--------K-----V---I--------L-I------------L--LI-Y-GG--
NARI-IVC5_NEM.J41      -Q--R-IS---DI--R---N--N---K--KD--A--S-DN--S--S--------R-----V---I--------L-L------------F--LT---RE--
NARI-IVC5-3_NEM.J2     -Q--R-IS---NI--R---N--N---K--KD--A--S-DN--S--S--------R-----V---I--------L-L------------F--LT---RE--
NARI-IVC5-3_NEM.J4     -Q--R-IS---NI--R---N--N---K--KD--A--S-DN--S--S--------R-----V---I--------L-L------------F--LT---RE--
NARI-IVC5-3_NEM.J5     -Q--R-IS---NI--R---N--N---K--KD--A--S-DN--S--S--------R-----V---I--------L-L------------F--LT---RE--
NARI-IVC5-3_NEM.J9     -Q--R-IS---NI--R---N--N---K--KD--A--S-DN--S--S--------R-----V---I--------L-L------------F--LT---RE--
NARI-IVC5-4_NEM.J16    -Q--R-IS---AT--R---N--N---K--KD--A--S-DN--S--S--------R-----V---I--------L-L------------F--LT---RE--
NARI-IVC5-4_NEM.J18    -Q--R-IS---DI--R---N--N---K--KD--A--S-DN--S--S--------R-----V---I--------L-L------------F--LT---RE--
NARI-IVC5-4_NEM.J22    -Q--K-IS---DI--R---N--N---K--KD--A--S-DN--S--S--------R-----V---I--------L-L------------F--PT---RE--
NARI-IVC5-4_NEM.J49    -Q--K-IS---DI--R---N--N---K--KD--A--S-DN--S--S--------R-----V---I--------L-L------------F--PT---RE--
NARI-IVC11_NEM.J25     -Q--R-IN---NT--R---E--S---E--KD--A--K-NN--N--N--------K-----V---I--------L-I------------F--LT---RE--
NARI-IVC11_NEM.J28     -Q--R-IN---NT--R---E--S---E--KD--A--K-NN--N--N--------K-----V---I--------L-I------------F--LT---RE--
NARI-IVC11-3_NEM.J3    -Q--R-IN---NT--R---E--S---E--KD--A--K-NN--N--N--------K-----V---I--------L-I------------F--PT---RE--
NARI-IVC11-3_NEM.J9    -Q--R-IN---NT--R---E--S---E--KD--A--K-NN--N--N--------K-----V---I--------L-I------------F--PT---RE--
NARI-IVC11-3_NEM.J16   -Q--R-IN---NT--R---E--S---E--KD--A--K-NN--N--N--------K-----V---I--------L-I------------F--PT---RE--
NARI-IVC11-5_NEM.J12   -Q--R-IN---NT--R---E--S---E--KD--A--K-NN--N--N--------K-----V---I--------L-I------------F--PT---RE--   700
```

Consensus	RlgrIEEEGGEQdXXRSvRLVsGFLAlaWDDLRsLCLFSYHRLRDfILVtaRvVELLGrsslXglqXGWeaLKYLgsLvqYWgleLKksAIsLLDtiAIX
NARI-IVC2-NEM.J8	-LGR--------DKT--V---S----LV-----S-----------F---AA-V-----RSSLKGLQK--TA----GS-VQ--GLE--KS--S---TV--A
NARI-IVC2_NEM.J9	-LGR--------DKT--V---S----LA-----S-----------F---AV-V-----HSSLKGLQK--TA----GS-VQ--GLE--KS--S---TV--A
NARI-IVC2-3_NEM.J4	-LGR--------DKT--V---S----LA-----S-----------F---AV-V-----HSSLKGLQK--TA----GS-VQ--GLE--KS--S---TI--A
NARI-IVC2-3_NEM.J7	-LGR--------DKT--V---S----LA-----S-----------F---AV-V-----HSSLKGLQK--TA----GS-VQ--GLE--KS--S---TI--A
NARI-IVC2-3_NEMJ.17	-LGR--------DKT--V---S----LA-----S-----------F---AV-V-----HSSLKGLQK--TA----GS-VQ--GLE--KS--S---TI--A
NARI-IVC2-3_NEM.J18	-LGR--------DKT--V---S----LA-----S-----------F---AV-V-----HSSLKGLQK--TA----GS-VQ--GLE--KS--S---TI--A
NARI-IVC2-5_NEM.J3	-LGR--------DKT--V---S----LA-----S-----------F---AA-V-----HRGWKGLQK-GTA----GS-VQ......KS--S---TI--A
NARI-IVC2-5_NEM.J11	-LGR--------DKT--V---S----LA-----S-----------F---AA-V-----HSSLKGLQK--TA----GS-VQ--GLE--KS--S---TI--A
NARI-IVC3_NEM.J16	-PGR--------DR**N**--I---S----LT-----S-----------L---TA-G-----HSSLKGLRL--DG----WN-LA--GRE--TS--N---TI--V
NARI-IVC3-3_NEM.J9	-PGR--------DR**N**--I---S----LS-----S-----------L---TA-G-----HSSLKGLRL--EG----WN-LA--GRE--TS--N---TI--V
NARI-IVC3-5_NEM.J25	-PGR--------DR**N**--I---S----LT-----S-----------L---TA-G-----HSSLKGLRL--EG----WN-LA--GRE--TS--N---TI--V
NARI-IVC3-5_NEM.J38	-PGR--------DR**N**--I---S----LS-----S-----------L---AA-G-----HSSLKGLRL--EG----WN-LA--GRE--TS--N---TI--V
NARI-IVC4_NEM.J2	-PGG--------DRA--V---N----LA-----N-----------L---TV-V-----RSSLRGLQR--EA----GS-VQ--GLE--KS--S---ST--V
NARI-IVC4_NEM.J22	-PGG--------DRA--V---N----LA-----N-----------L---TV-V-----RSSLRGLQR--EA----GS-VQ--GLE--KS--S---ST--V
NARI-IVC4_NEM.J27	-PGG--------DRA--V---N----LA-----N-----------L---TV-V-----RSSLRGLQR--EA----GS-VQ--GLE--KS--S---ST--V
NARI-IVC4-2_NEM.J41	-PGG--------DRA--V---N----PA-----N-----------L---TV-V-----RSSLRGLQR--EA----GS-VQ--GLG--KS--S---ST--V
NARI-IVC4-2_NEM.J45	-LGG--------DRA--V---N----LA-----N-----------L---TV-V-----R.......R--EA----GS-VQ--GLE--KS--S---ST--V
NARI-IVC4-2_NEM.J42b	-LGG--------DRA--V---N----LA-----N-----------L---TV-V-----R.......R--EA----GS-VQ--GLE--KS--S---ST--V
NARI-IVC4-2_NEM.J45b	-LGG--------DRA--V---N----LA-----N-----------L---TV-V-----R.......R--EA----GS-VQ--GLE--KS--S---ST--V
NARI-IVC4-2_NEM.J46b	-LGG--------DRA--V---N----LA-----N-----------L---TV-V-----R.......R--EA----GS-VQ--GLE--KS--S---ST--V
NARI-IVC4-2_NEM.J47b	-PGG--------DRA--V---N----LA-----N----------GL---TV-V-----R.......R--EA----GS-VQ--GLE--KS--S---ST--V
NARI-IVC4-5_NEM.J5	-PGG--------DRA--V---N----LA-----N-----------L---TV-V-----R.......R--EA----GS-VQ--GLE--KS--S---ST--V
NARI-IVC5_NEM.J41	-LGR--------GND--I---S----LA-----S-----C-----F---AA-V-----RSSLQGLQK--EA----GS-VQ--VLE--KS--S---TI--T
NARI-IVC5-3_NEM.J2	-LGR--------GND--V---S----LA-----S-----------F---AA-V-----RSSLQGLQK--EA----GS-VQ--VLE--RS--S---TI--T
NARI-IVC5-3_NEM.J4	-LGR--------GND--V---S----LA-----S-----------F---AA-V-----RSSLQGLQK--EA----GS-VQ--VLE--RS--S---TI--T
NARI-IVC5-3_NEM.J5	-LGR--------GND--V---S----LA-----S-----------F---AA-V-----RSSLQGLQK--EA----GS-VQ--VLE--RS--S---TI--T
NARI-IVC5-3_NEM.J9	-LGR--------GND--V---S----LA-----S-----------F---AA-V-----RSSLQGLQK--EA----GS-VQ--VLE--RS--S---TI--T
NARI-IVC5-4_NEM.J16	-LGR--------GND--V---S----LA-----S-----------F---AA-V-----RSSLQGLLK--EA----GS-VQ--VLE--KS--S---TI--T
NARI-IVC5-4_NEM.J18	-LGR--------GND--V---S----LA-----S-----------F---AA-V-----RSSLQGLLK--EA----GS-VQ--VLE--KS--S---TI--T
NARI-IVC5-4_NEM.J22	-LGR--------GND--V---S----LA-----S-----------F---TA-V-----RSSLQGLLK--EA----GS-VQ--VLE--KS--S---TI--T
NARI-IVC5-4_NEM.J49	-LGR--------GND--V---S----LA-----S-----------F---TA-V-----RSSLQGLLK--EA----GS-VQ--VLE--KS--S---TI--T
NARI-IVC11_NEM.J25	-LRR--------DNV--V---T----IA-----N-----------F---TA-V-----HSSLRGLQR--KA----GS-VQ--GLE--KG--S---TI--A
NARI-IVC11_NEM.J28	-LRR--------DNV--V---T-L--IA-----N-----------F---TA-V-----HSSLRGLQR--KA----GS-VQ--GLE--KG--C---TI--A
NARI-IVC11-3_NEM.J3	-LRR--------DNV--V---T----IA-----N-----------F---TA-V-----HSSLRGLQG--EA----GS-VQ--GLE--KG--S---TI--A
NARI-IVC11-3_NEM.J9	-LRR--------DNV--V---T----IA-----N-----------F---TA-V-----HSSLRGLQR--EA----GS-VQ--GLE--KG--S---TI--A
NARI-IVC11-3_NEM.J16	-LRR--------DNV--V---T----IA-----N-----------F---TA-V-----HSSLRGLQR--EA----GS-VQ--GLE--KG--S---TI--A
NARI-IVC11-5_NEM.J12	-LRR----D---DNV--V---T----IA-----N-----------F---TA-V-----HSSLRGLQR--TA----GS-VQ--GLE--KG--S---TI--A 800

```
Consensus                VAegTDRiieXXqricRaiXNiPrRIRQGlEAaLQ
NARI-IVC2-NEM.J8         --EG---IIEFFQRLC-AIC-I-R-----F--A--
NARI-IVC2_NEM.J9         --EG---IIEFSQRLC-AIC-I-R-----F--A--
NARI-IVC2-3_NEM.J4       --EG---IIEFLQRLC-AIC-I-R-----F--A--
NARI-IVC2-3_NEM.J7       --EG---IIEFLQRLC-AIC-I-R-----F--A--
NARI-IVC2-3_NEMJ.17      --EG---IIEFLQRLC-AIC-I-R-----F--A--
NARI-IVC2-3_NEM.J18      --EG---IIEFLQRLC-AIC-I-R-----F--A--
NARI-IVC2-5_NEM.J3       --EG---IIEFLRRLC-AIC-I-R-----F--A--
NARI-IVC2-5_NEM.J11      --EG---IIEFLRRLC-AIC-I-R-----F--A--
NARI-IVC3_NEM.J16        --GW---LIEIGQGIC-TIR-I-R-----L--T--
NARI-IVC3-3_NEM.J9       --GW---LIEIGQGIC-AIR-V-R-----L--T--
NARI-IVC3-5_NEM.J25      --GW---LIEIGQRIC-AIR-V-R-----L--T--
NARI-IVC3-5_NEM.J38      --GW---LIEIGQGIC-AIR-V-R-----L--T--
NARI-IVC4_NEM.J2         --EG---IIKAVQGIC-TIR-I-T-----L--A--
NARI-IVC4_NEM.J22        --EG---IIKAVQGIC-TTR-I-T-----L--A--
NARI-IVC4_NEM.J27        --EG---IIKAVQGIC-TIR-I-T-----L--A--
NARI-IVC4-2_NEM.J41      --EG---IIKAVQGIC-TIR-I-T-----L--A--
NARI-IVC4-2_NEM.J45      --EG---IIKAVQGIC-TIR-I-T-----L--A--
NARI-IVC4-2_NEM.J42b     --EG---IIKAVQGIC-TIR-I-T-----L--A--
NARI-IVC4-2_NEM.J45b     --EG---VIEAVQGIC-TIR-I-T-----L--A--
NARI-IVC4-2_NEM.J46b     --EG---IIKAVQGIC-TIR-I-T-----L--A--
NARI-IVC4-2_NEM.J47b     --EG---IIKAVQGIC-TIR-I-T-----L--A--
NARI-IVC4-5_NEM.J5       --EG---IIGAVQGIC-TIR-I-T-----L--A--
NARI-IVC5_NEM.J41        --EG---IIEVIQRTC-VIY-I-R-----L--A--
NARI-IVC5-3_NEM.J2       --EG---IIEVIQRTC-VIY-I-R-----L--A--
NARI-IVC5-3_NEM.J4       --EG---IIEVIQRTC-VIY-I-R-----L--A--
NARI-IVC5-3_NEM.J5       --EG---IIEVIQRTC-VIY-I-R-----L--A--
NARI-IVC5-3_NEM.J9       --EG---IIEVIQRTC-VIY-I-R-----L--A--
NARI-IVC5-4_NEM.J16      --EG---IIEGIQRTC-AIY-I-R-----L--A--
NARI-IVC5-4_NEM.J18      --EG---IIEGIQRTC-AIY-I-R-----L--A--
NARI-IVC5-4_NEM.J22      --EG---IIEGIQRTC-AIY-I-R-----L--A--
NARI-IVC5-4_NEM.J49      --EG---IIEGIQRTC-AIY-I-R-----L--A--
NARI-IVC11_NEM.J25       --EG---IIELVQRIC-AFC-I-R-----F--A--
NARI-IVC11_NEM.J28       --EG---IIELVQRIC-AFC-I-R-----F--A--
NARI-IVC11-3_NEM.J3      --EG---IVELVQRIG-AFC-I-R-----F--A--
NARI-IVC11-3_NEM.J9      --EG---IVELVQRIG-AFC-I-R-----F--A--
NARI-IVC11-3_NEM.J16     --EG---IVELVQRIG-AFC-I-R-----F--A--
NARI-IVC11-5_NEM.J12     --EG---IIELGQRIC-AFC-I-R-----F--A-- 800
```
