# Supplementary material for: Variations in autologous neutralization and CD4 dependence of b12 resistant HIV-1 clade C env clones obtained at different time points from antiretroviral naïve Indian patients with recent infection
Source: Retrovirology. 2010 Sep 22;7:76. doi: 10.1186/1742-4690-7-76 (PMC2955667; doi:10.1186/1742-4690-7-76)
Supplement: Additional file 2 — Table S1. 2F5 and 4E10 minimum motifs in MPER domain in patient Envs and their corresponding sensitivities to 2F5 and 4E10 monoclonal antibodies. [file 1742-4690-7-76-S2.PDF]

Supplementary Table S1

Env clones	2F5 recognition motif	2F5 Sensitivity	4E10 recognition motif	4E10 Sensitivity
2.J8	AL**DRW**G	RESISTANT	N**WFNI**T	SENSITIVE
2.J9	AL**DRW**G	RESISTANT	N**WFNI**T	SENSITIVE
2-3.J4	AL**DRW**G	RESISTANT	N**WFNI**T	SENSITIVE
2-3.J7	AL**DRW**G	RESISTANT	N**WFN**IT	SENSITIVE
2-3.J17	GL**DRW**G	RESISTANT	N**WFNI**T	SENSITIVE
2-3.J18	AL**DRW**G	RESISTANT	N**WFNI**T	SENSITIVE
2-5.J3	AL**DRW**G	RESISTANT	N**WFNI**T	SENSITIVE
2-5.J11	AL**DRW**G	RESISTANT	N**WFNI**T	SENSITIVE
3.J16	EL**DSW**K	RESISTANT	S**WFDI**T	SENSITIVE
3-3.J9	EL**DKW**K	RESISTANT	S**WFDI**T	SENSITIVE
3-5.J25	EL**DKW**K	RESISTANT	S**WFDI**T	SENSITIVE
3-5.J38	**ELDSW**K	RESISTANT	S**WFDI**T	RESISTANT
4.J2	AL**DSW**N	RESISTANT	N**WFGI**T	RESISTANT
4.J22	AL**DSW**N	RESISTANT	N**WFGI**T	SENSITIVE
4.J27	AL**DSW**N	RESISTANT	N**WFGI**T	SENSITIVE
4-2.J41	AL**DSW**N	RESISTANT	N**WFDI**T	SENSITIVE
4-2.J45	AL**DSW**N	RESISTANT	N**WFDI**T	SENSITIVE
4-2.J42b	AL**DSW**N	RESISTANT	N**WFG**IT	SENSITIVE
4-2.J45b	AL**DSW**N	RESISTANT	N**WFDI**T	SENSITIVE
4-2.J46b	AL**DSW**N	RESISTANT	N**WFGI**T	RESISTANT
4-2.J47b	AL**DSW**N	RESISTANT	N**WFG**IT	SENSITIVE
4-5.J5	AL**DSW**N	RESISTANT	N**WFDI**T	RESISTANT
5.J41	AL**DSW**D	RESISTANT	S**WFS**IT	SENSITIVE
5-3.J2	AL**DSW**D	RESISTANT	S**WFSI**T	SENSITIVE
5-3.J4	AL**DSW**D	RESISTANT	S**WFSI**T	RESISTANT
5-3.J5	AL**DSW**D	RESISTANT	S**WFSI**T	SENSITIVE
5-3.J9	AL**DSW**D	RESISTANT	S**WFSI**T	RESISTANT
5-4.J16	AL**DSW**D	RESISTANT	S**WFSI**T	SENSITIVE
5-4.J18	AL**DSW**D	RESISTANT	S**WFSI**T	SENSITIVE
5-4.J22	AL**DSW**D	RESISTANT	S**WFSI**T	SENSITIVE
5-4.J49	AL**DSW**D	RESISTANT	S**WFSI**T	SENSITIVE
11.J25	AL**DKW**N	RESISTANT	N**WFNI**T	SENSITIVE
11.J28	AL**DKW**N	RESISTANT	N**WFNI**T	SENSITIVE
11-3.J3	AL**DKW**N	RESISTANT	N**WFNI**T	SENSITIVE
11-3.J9	AL**DKW**N	RESISTANT	N**WFNI**T	SENSITIVE
11-3.J16	AL**DKW**N	RESISTANT	N**WFNI**T	SENSITIVE
11-5.J12	AL**DKW**N	RESISTANT	N**WFNI**T	SENSITIVE
