# Supplementary figures and images for: Variations in autologous neutralization and CD4 dependence of b12 resistant HIV-1 clade C env clones obtained at different time points from antiretroviral naïve Indian patients with recent infection
Source: Retrovirology. 2010 Sep 22;7:76. doi: 10.1186/1742-4690-7-76 (PMC2955667; doi:10.1186/1742-4690-7-76)

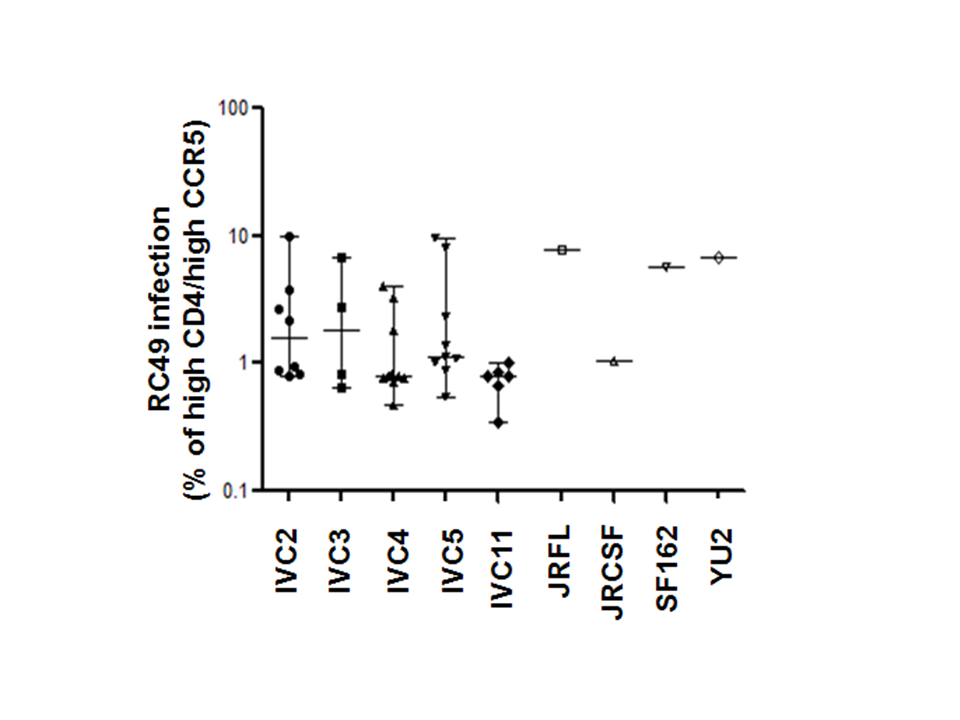

Supplement: Additional file 3 — Figure S2. Variations in CD4 dependence of patient Envs obtained at different time points in each patient. Note that the bar represents the median percentage infectivity of pseudoviruses to RC49 cells expressing low CD receptors. [file 1742-4690-7-76-S3.TIFF]
